# Potential active ingredients and mechanisms of Shufeitie ointment in the treatment of chronic obstructive pulmonary disease by integrating transdermal chemistry and network pharmacology

**DOI:** 10.3389/fmed.2025.1605372

**Published:** 2025-07-08

**Authors:** Jianing Sun, Di Zhang, Panpan Wang, Junhan Shi, Tan Xue, Panpan Zhang, Qi Wang, Xinjing Gui, Suyun Li, Jiansheng Li, Xuelin Li, Ruixin Liu

**Affiliations:** ^1^Department of Pharmacy, The First Affiliated Hospital of Henan University of Chinese Medicine, Zhengzhou, Henan, China; ^2^School of Pharmacy, Henan University of Chinese Medicine, Zhengzhou, Henan, China; ^3^Henan Province Engineering Research Center for Clinical Application, Evaluation and Transformation of Traditional Chinese Medicine, Zhengzhou, Henan, China; ^4^Henan Key Laboratory for Clinical Pharmacy of Traditional Chinese Medicine, Zhengzhou, Henan, China; ^5^Co-construction Collaborative Innovation Center for Chinese Medicine and Respiratory Diseases by Henan & Education Ministry of P.R. China, Henan University of Traditional Chinese Medicine, Zhengzhou, Henan, China; ^6^Respiratory Department of the First Affiliated Hospital of Henan University of Chinese Medicine, Zhengzhou, Henan, China; ^7^Engineering Research Center of Ministry of Education, Pharmaceutical and New Drug Development of Traditional Chinese Medicine, Beijing, China

**Keywords:** Shufeitie ointment, chronic obstructive pulmonary disease, UHPLC-Q-orbitrap/MS, external preparations, Franz diffusion cell, network pharmacology

## Abstract

**Purpose:**

This study aims to identify the transdermal penetration components of Shufeitie ointment (SFTOT) and investigate the potential active components and mechanisms through which SFTOT exerts its effects on Chronic Obstructive Pulmonary Disease (COPD).

**Methods:**

An *in vitro* permeation test (IVPT) of SFTOT was conducted using a modified Franz diffusion cell method. Ultra-high-performance liquid chromatography-quadrupole/electrostatic field orbitrap high-resolution mass spectrometry (UHPLC-Q-Orbitrap/MS) was employed to analyze data from the transdermal receiving solution, enabling comprehensive identification of the components that permeate through the skin. To predict the potential mechanisms by which SFTOT may treat COPD, network pharmacology was used to construct a component-target-collaterals network. Additionally, molecular docking was applied to verify the interactions between the potential transdermal active components of SFTOT and the core targets.

**Results:**

Using UHPLC-Q-Orbitrap/MS, we identified 129 transdermal permeation components in SFTOT. Network pharmacology analysis revealed 222 common targets between SFTOT and COPD. The primary active components were predicted to be luteolin, kaempferol, quercetin, 7-O-methylluteolin, apigenin, ferulic acid, palmitic acid, inapinic acid, 6-shogaol, and myristic acid. These components were primarily enriched in the AGE-RAGE, TNF, PI3K-Akt, and MAPK signaling pathways. Protein–protein interaction (PPI) analysis identified TNF, ALB, AKT1, EGFR, and CASP3 as core targets. Molecular docking results showed that 72% of component-target interactions had a binding energy of < −5.0 kcal/mol, indicating strong binding activity. Among these, apigenin exhibited the lowest binding energy with EGFR and consistently lower binding energies with other core targets compared to the other components. This suggests that apigenin may play a key role in treatment.

**Conclusion:**

High-resolution liquid chromatography-mass spectrometry effectively identified the transdermal penetration components of SFTOT, providing a foundation for further screening of key active compounds. Our findings suggest that SFTOT may alleviate COPD by downregulating TNF, ALB, AKT1, EGFR, and CASP3 while inhibiting inflammatory mediator release through the AGE-RAGE, TNF, PI3K-Akt, and MAPK signaling pathways. These effects may help reduce COPD-related symptom clusters. Notably, apigenin appears to be a crucial bioactive component in the prevention and treatment of COPD.

## 1 Introduction

Chronic Obstructive Pulmonary Disease (COPD) is a progressive respiratory disorder characterized by airflow limitation. Although its pathogenesis remains incompletely understood, multiple factors contribute to its development, including smoking, occupational exposure to dust and chemicals, air pollution, infections, and an imbalance between proteases and antiproteases (1). Additionally, autonomic dysfunction, heightened airway responsiveness, and chronic inflammation play crucial roles in disease progression (2). The primary clinical symptoms of COPD include cough, sputum production, and dyspnea (3). As the disease advances, patients may develop respiratory failure, pulmonary hypertension, and pulmonary heart disease, which can become life-threatening. COPD is associated with three major burdens: high prevalence, high mortality, and substantial economic costs. According to the Global Burden of Disease Study (2019), an estimated 212.3 million people worldwide had COPD (4). In 2021, the World Health Organization reported that COPD caused 3.5 million deaths, accounting for approximately 5% of total global mortality. As the fourth leading cause of death worldwide (5), COPD is a critical public health concern requiring urgent attention.

In recent years, Chinese medicine has made significant strides in treating COPD. In Traditional Chinese Medicine (TCM), COPD falls under the categories of lung distension, cough, and asthma. TCM treatment approaches include both internal and external therapies. Internal treatments primarily involve the oral administration of traditional Chinese medicinal decoctions and patent medicines, such as Liuwei Buqi Decoction (6), Shiwei Longdanhua Decoction (7), Jianpi Yifei II Granules (8), and Bufei Jianpi Granules (9). These therapies have been shown to effectively alleviate chronic inflammation and enhance respiratory function in COPD patients. The underlying mechanism may involve the inhibition of inflammatory responses and the reduction of airway epithelial cell apoptosis (10). External treatments include application therapy, acupuncture, and fumigation therapy, all of which have demonstrated positive effects in alleviating COPD symptoms and promoting rehabilitation (11–13). Acupoint application therapy (14, 15), in particular, is a distinctive treatment modality in TCM, grounded in traditional theories and ancient clinical experience. It integrates the concepts of acupoints, meridians, and medicinal substances. By stimulating acupoints and facilitating drug absorption through the skin, this therapy promotes the circulation of qi and blood, unblocks meridians, and helps regulate the zang-fu organs. This approach has shown significant preventive and therapeutic effects on various chronic respiratory diseases, leveraging both the meridian and medicinal properties. The mechanism of action is thought to involve immune regulation and the inhibition of airway inflammation and remodeling.

Shufeitie ointment [SFTOT; Approval No. Yuyao Zhizi Z20120014 (Zheng)] is an external preparation developed by the First Affiliated Hospital of Henan University of Traditional Chinese Medicine. It was formulated based on over 40 years of clinical experience and integrates modern pharmacology and preparation technology. As a novel external treatment for COPD, SFTOT consists of seven traditional Chinese medicinal ingredients, including Sinapis Semen (*Sinapis alba* L., or *Brassica juncea* (L.)Czern. et Coss., Jiezi), Genkwa Flos (*Daphne genkwa Sieb*. et Zucc., Cuyuanhua), Corydalis Rhizoma (*Corydalis yanhusuo* W. T. Wang., Cuyanhusuo), Cinnamomi Cortex (*Cinnamomum cassia* Presl., Rougui), Semen Zanthoxyli (*Zanthoxylum bungeanum* Maxim or *Zanthoxylum schinifolium* Sieb.et Zucc., Jiaomu), Asari Radix Et Rhizoma (*Asarum heterotropoides* Fr. Schmidt var. mandshuricum (Maxim.) Kitag., or *Asarum sieboldii* Miq.var. seoulense Nakai., or *Asarum sieboldii* Miq., Xixin), Zingiberis Rhizoma (*Zingiber officinale* Rosc., Ganjiang). Modern pharmacological studies have demonstrated that SFTOT reduces inflammatory cytokines, including tumor necrosis factor (TNF), thereby alleviating inflammation in the airways and lung tissue. Additionally, it significantly increases immunoglobulin levels in COPD rats and modulates T lymphocyte expression, which helps improve lung function and mitigate pathological lung tissue damage (16). These findings align with the pathological and mechanistic understanding of COPD. Clinical studies have shown that the control group was treated with western medicine, and the experimental group was treated with SFTOT acupoint external application combined with western medicine. Each group included 38 patients with acute exacerbation of chronic obstructive pulmonary disease in winter. The results showed that the total effective rate of the experimental group was 94.74%, which was significantly higher than 78.95% of the control group (*p* < 0.05). The improvement of respiratory function in the experimental group was significantly better than that in the control group (*p* < 0.05). There was no significant difference in the incidence of adverse reactions between the two groups (*p* > 0.05) (17). Further observation of patients with stable COPD found that the selection of bilateral Feishu, bilateral Shenshu, Dazhui, Tanzhong and other acupoints for SFTOT application treatment, 8 ~ 12 h application time can effectively alleviate the symptoms of acute exacerbation of COPD, the clinical effect is significant. The study also suggests that the foaming phenomenon after application may be positively correlated with the clinical effect (18, 19). However, systematic and comprehensive studies on the active components and pharmacological mechanisms of SFTOT in COPD treatment remain limited. Therefore, this study aims to identify the transdermal active components of SFTOT and elucidate its mechanism of action against COPD.

TCM compounds exhibit therapeutic effects through multi-component, multi-channel, and multi-target mechanisms. Understanding the pharmacodynamic material basis of these compounds is essential for their development and for elucidating their pharmacological mechanisms. To evaluate the efficacy of SFTOT in COPD treatment, we employed a Franz diffusion cell system combined with UHPLC-Q-Orbitrap/MS technology to investigate its skin permeability. Additionally, we used network pharmacology and molecular docking techniques to identify potential active components and explore their mechanisms of action. This approach overcomes the limitations of previously unknown components and mechanisms of SFTOT, providing valuable insights for new drug development.

## 2 Materials and methods

### 2.1 Chemicals and reagents

We sourced all decoction pieces from Henan Zhongyi Pharmaceutical Co., Ltd. (Zhengzhou, China) and Shi Junhan, Deputy Director of the First Affiliated Hospital of Henan University of Traditional Chinese Medicine, verified their authenticity. The ingredients included *Sinapis Semen* (JieZi, JZ; No: J5772113), *Corydalis Rhizoma* (CuYanHuSuo, CYHS; No: 230304), *Zingiberis Rhizoma* (GanJiang, GJ; No: 221201), *Asari Radix Et Rhizoma* (XiXin, XX; No: 20031316), *Cinnamomi Cortex* (RouGui, RG; No: 22120207), *Semen Zanthoxyli* (JiaoMu, JM; No: 2209211), and *Genkwa Flos* (CuYuanHua, CYH; No: 211102). Sodium carboxymethyl cellulose (No: B2215321) and glycerol (No: D2213012) from Shanghai Aladdin Biochemical Technology Co., Ltd. (Shanghai, China). Ethanol (No: 20220908) from Kangbao Biochemical Technology Co., Ltd. (Shandong, China). Laurocapram (No: 20210302) from Xinxiang Gaojin Pharmaceutical Co., Ltd. (Henan, China). 0.9% NaCl aqueous solution (No: A22120801A) from Henan Kelun Pharmaceutical Co., Ltd. (Henan, China).

Chromatographic acetonitrile and Pierce™ formic acid (chromatographic grade) were purchased from Thermo Fisher (Massachusetts, United States). Chromatographic grade methanol was obtained from Shanghai Aladdin Biochemical Technology Co., Ltd. (Shanghai, China). Ultrapure water was supplied by the Elemental 18,120 Molecular ultrapure water system from Shanghai Moller Scientific Instrument Co., Ltd. (Shanghai, China). The back skin of SPF BALB/C-nu mice was obtained from Yantai Raphael Biotechnology Co., Ltd. (Shandong, China) under License No. SCXK (Lu) 2022–0006 and Animal Certificate No. 370726221101453317.

### 2.2 Shufeitie ointment preparation

Following the established preparation method for SFTOT at the hospital (20), volatile oils were extracted from JieZi (JZ), GanJiang (GJ), XiXin (XX), JiaoMu (JM), and RouGui (RG) through steam distillation. The resulting distilled water solution was reserved for later use. CuYanHuSuo (CYHS) and CuYuanHua (CYH) were added to the remaining dregs, followed by the addition of 10 times the amount of 70% ethanol. The mixture was refluxed and extracted three times, each for 1 h. The three extracts were combined, filtered, and the filtrate was mixed with the previously prepared aqueous solution. The ethanol was removed by vacuum distillation, and the resulting concentrated mixture was turned into a clear paste and set aside. Next, sodium carboxymethyl cellulose was added to the paste and allowed to swell overnight. After thorough stirring, volatile oil, glycerol, and azone were incorporated. The mixture was stirred to ensure homogeneity, defoamed, and the final preparation was obtained.

### 2.3 *In vitro* penetration test

#### 2.3.1 Preparation of the isolated skin

The previously prepared mouse skin was removed from storage at −20°C and placed in normal saline at room temperature for 15 min to thaw. For each mouse, three skin samples were selected and cut into three pieces, each corresponding to the area of the diffusion pool.

#### 2.3.2 Weighing transdermal drug samples

Three portions of Shufeitie ointment (SFTOT) were weighed in parallel, with each portion weighing approximately 1.03 ± 0.01 g, 1.02 ± 0.01 g, and 1.02 ± 0.01 g, respectively.

#### 2.3.3 Collection of samples

The *in vitro* transdermal absorption experiment of SFTOT was conducted using a Franz diffusion cell system (Model #TPY-2, Shanghai Huanghai Drug Control Instrument Co., Ltd., China). The system consisted of a heating cycle, a temperature control system, magnetic stirring, a vertical diffusion cell with a 25 mL receptor capacity, and an effective diffusion area of 3.14 cm^2^. The upper chamber served as the diffusion cell, while the lower chamber acted as the receiving cell. The isolated skin, as described in section 2.3.1, was fixed between the diffusion pool and the receiving pool, with the stratum corneum facing the diffusion pool. The drug, as weighed in Section 2.3.2, was evenly applied to the stratum corneum of the rat skin, and the diffusion pool was sealed with a sealing film. A magnet was placed in the receiving pool, which contained 7 mL of normal saline, preheated to 32°C and degassed by ultrasound. The liquid surface was in contact with the inner layer of the skin to avoid bubbles between the rat skin and the receiving liquid. If necessary, additional normal saline at the same temperature was added, and any bubbles in the inclined diffusion pool were removed. After setup, the system was maintained at a constant temperature of 32 ± 1°C using a preheated water bath, and stirring was initiated at a constant speed of 300 rpm. Samples were taken at 1, 2, 4, 6, 8, and 12 h and placed into 100 mL centrifuge tubes. An equal volume of degassed homothermal receiving liquid was immediately added to each sample.

### 2.4 Analysis of Shufeitie ointment transdermal permeation components via UHPLC-Q-Orbitrap/MS

#### 2.4.1 Testing conditions

Chromatographic separation was performed using an Ultimate 3,000 ultra-high-performance liquid chromatography (UHPLC) system (Thermo, United States) equipped with a Hypersil GOLD™ VANQUISH™ C_18_ column (2.1 mm × 100 mm, 1.9 μm). The column was maintained at 40°C, and the flow rate was set to 0.2 mL/min. The mobile phase consisted of 0.1% formic acid in water (A) and acetonitrile (B), with a gradient elution as follows: 0–7 min, 10–40% B; 7–13 min, 40–80% B; 13–25 min, 80–95% B; 25–25.1 min, 95–10% B; 25.1–30 min, 10–10% B. The injection volume was 5 μL.

Mass spectrometry detection was performed using an Orbitrap Exploris 240 MS (Thermo, United States). Electrospray ionization (ESI) was carried out in both positive ion (ESI^+^) and negative ion (ESI^−^) modes. The optimized ESI-MS parameters were as follows: spray voltages of 3,500 V (positive mode) and 3,000 V (negative mode), sheath gas flow rate of 35 Arb, auxiliary gas flow rate of 12 Arb, and a capillary temperature of 350°C. Full Scan/dd-MS^2^ scanning mode was used with a Full MS resolution of 60,000 and dd-MS^2^ resolution of 15,000. The collision energy was set to 20, 40, and 60 eV, and the scanning range was m/z 70–1,050.

#### 2.4.2 Treatment of samples

At 6 time points, 3 mL of the transdermal receiving solution, as described in Section 2.3.3, was pooled. The solution was evaporated to dryness at 45°C using a centrifugal concentrator, then reconstituted in 1 mL of methanol. The mixture was filtered through a 0.22 μm filter membrane, centrifuged at 14,000 rpm for 10 min at 20°C, and the supernatant was collected after two centrifugation steps for further analysis.

#### 2.4.3 Data analysis

Following data collection, the raw data from the UHPLC-Orbitrap Exploris 240 system were imported into Compound Discoverer 3.0 (CD) software for preliminary analysis. This included peak extraction, peak alignment, retention time correction, and extraction peak area determination. The identification of unknown compounds was performed by matching the fragment ions with the mzCloud database,^1^ mzVault database, and ChemSpider database.^2^ The results were further compared with relevant literature to ensure accurate identification of the chemical components in the SFTOT transdermal receiving solution.

### 2.5 Network pharmacology analysis

#### 2.5.1 Screening of drug targets and collection of disease targets

All identified active compounds in Shufeitie ointment (SFTOT) capable of penetrating the skin were uploaded to the SwissTargetPrediction,^3^ PharmMapper,^4^ and SEA^5^ databases for target prediction. To maximize the number of potential targets, we integrated results from all three databases. We selected targets with Probability ≥ 0.2 in SwissTargetPrediction, NormFit ≥ 0.8 in PharmMapper, and MaxTc ≥ 0.4 in SEA. After compiling the data, we removed duplicate entries to establish the SFTOT target library. Next, we identified human target genes associated with Chronic Obstructive Pulmonary Disease (COPD) using the OMIM,^6^ DrugBank,^7^ and GeneCards^8^ databases, using “chronic obstructive pulmonary disease” as the search term. For GeneCards, we applied a GeneScore > 10 threshold to refine our selection. We compiled the retrieved data, removed duplicates, and established the COPD target library.

To standardize gene names, we used the UniProt database^9^ to convert SFTOT and COPD target names into their standardized gene forms. We then identified overlapping targets between the SFTOT target library and the COPD target library, clarifying the interaction between prescription-related and disease-related targets. This intersection formed the final target library, representing potential SFTOT targets for COPD treatment.

#### 2.5.2 Constructing protein–protein interaction networks

The SFTOT-COPD intersection targets identified in section 2.5.1 were uploaded to the STRING database.^10^ We selected *Homo sapiens* as the biological species and set the minimum required interaction score to medium confidence (0.4). All other parameters were kept at their default settings. After screening the targets, we excluded unrelated ones, exported the data as a TSV file, and constructed the PPI network using Cytoscape (version 3.7.1) software to predict interactions between the targets. To analyze network topology, we employed the CytoNAC plug-in to calculate the closeness centrality (CC) and betweenness centrality (BC) of each node. Based on these results, we selected the key targets.

#### 2.5.3 Gene ontology and Kyoto encyclopedia of genes and genomes signaling pathway enrichment analysis

To further explore the mechanism of SFTOT, we imported the selected SFTOT-COPD intersection targets into the DAVID database^11^ and restricted the target gene list to human genes. We performed GO biological function enrichment analysis and KEGG pathway enrichment analysis (21). GO analysis was divided into three categories: cellular component (CC), biological process (BP), and molecular function (MF). We sorted the results by *p*-value and visualized the top 10 GO terms and top 20 KEGG pathways as bubble plots.

##### 2.5.4 Construction of the component-target-pathway network model

The SFTOT-COPD intersection targets enriched through KEGG analysis in section 2.5.3 and the SFTOT-COPD targets identified in section 2.5.1 were uploaded to Cytoscape 3.7.1^12^ to construct a component-target-pathway network. We calculated network topology parameters and identified the key active components of SFTOT involved in COPD intervention based on these parameters and relevant literature.

### 2.6 Molecular docking

We obtained the three-dimensional crystal structure of potential targets from the RCSB PDB database^13^ and saved them in PDB format. The three-dimensional structures of active compounds were retrieved from PubChem, saved as SDF files, and converted to PDB format using Open Babel 2.4.1 (22). We imported the PDB files of target proteins and active compounds into AutoDock 1.5.7^14^ for preprocessing, including water removal and hydrogenation. We designated the target protein as the receptor and the active compound as the ligand. Using the receptor protein’s grid box coordinates and size, we performed molecular docking to generate receptor-ligand binding results. This allowed us to calculate binding energy values, which we used to assess the stability of interactions between active components and potential targets, as well as to validate the reliability of the network pharmacology predictions. We visualized the docking results using LigPlot software.

## 3 Results

### 3.1 Qualitative study of Shufeitie ointment chemical constituents in transdermal permeation via UHPLC-Q-Orbitrap/MS

**Figure 1:**
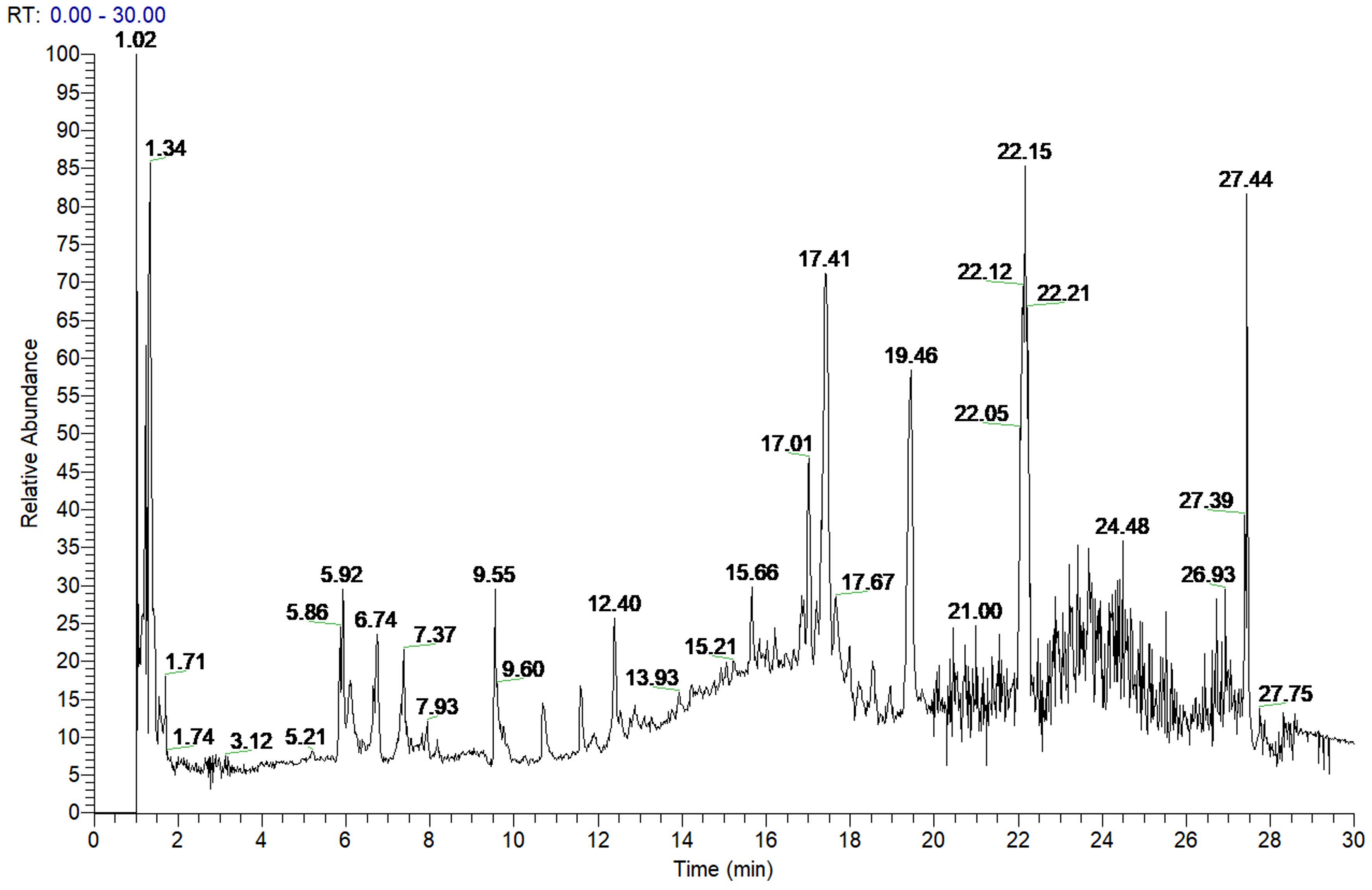
SFTOT transdermal component positive ion pattern.

**Figure 2:**
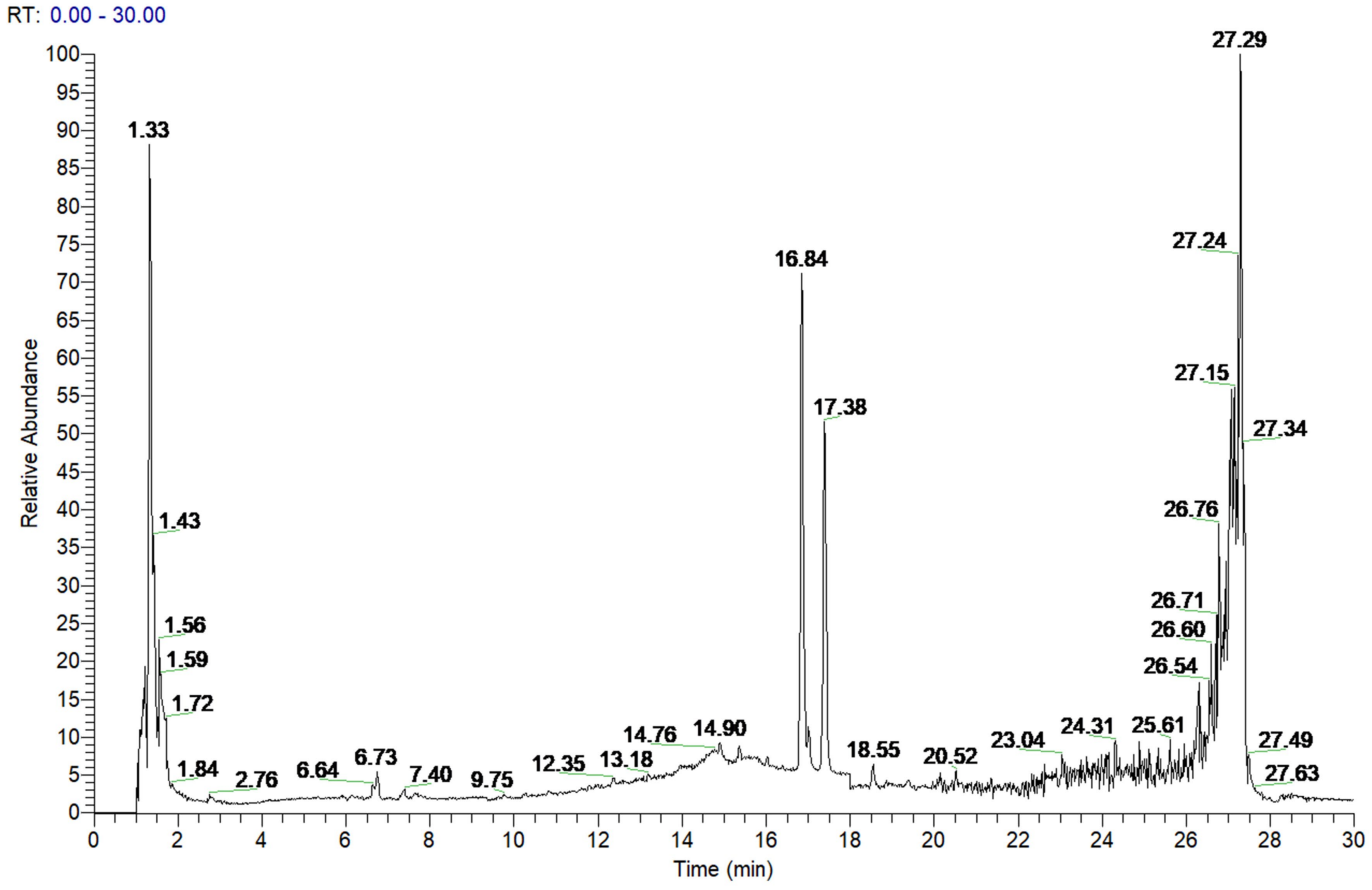
SFTOT transdermal component negative ion pattern.

To investigate the active components of SFTOT against Chronic Obstructive Pulmonary Disease (COPD), we obtained the transdermal receiving solution using a Franz diffusion cell. We rapidly separated and analyzed the compounds using UHPLC-Q-Orbitrap/MS technology. The scanning mode used was Full scan/data-dependent secondary scan (Full MS/dd MS^2^). The total ion chromatogram of the transdermal components of SFTOT, analyzed in both positive and negative ion modes, is shown in Figures 1, 2.

Using the high-resolution mass spectrometry data, we derived possible molecular formulas, ensuring that the mass spectrometry deviation remained within 5 × 10^−6^. With the assistance of Compound Discoverer 3.0 software and the unknown identification database, we rapidly identified the target compounds. This identification was based on accurate relative molecular mass, secondary mass spectrometry fragmentation patterns, and corroborated with both domestic and international literature. Ultimately, we characterized a total of 129 chemical components, including: 12 components from JieZi (JZ), 18 components from CuYuanHua (CYH), 20 components from CuYanHuSuo (CYHS), 19 components from GanJiang (GJ), 14 components from XiXin (XX), 11 components from RouGui (RG), 23 components from JiaoMu (JM), and 12 common components from various traditional Chinese medicines. The names, molecular formulas, and secondary fragments of these compounds are listed in Table 1. These compounds are considered the active ingredients of SFTOT, which may exert pharmacological effects through the skin.

**Table 1:** Identification of the common phytochemical components in SFTOT diffusion sample.

No.	t_R_/min	Compounds	Formula	Theoretical value(m/z)	Observed value(m/z)	Error(PPM)	Ion mode	Fragment ions (m/z)	Source
1	1.241	Lysine	C_6_ H_14_ N_2_ O_2_	146.1055	147.1128	−0.05	[M + H]^+^	147.1134;130.0862;84.0806;56.0495	JM
2	1.249	Arginine	C_6_ H_14_ N_4_ O_2_	174.1116	175.1189	−0.19	[M + H]^+^	175.1190;158.0923;116.0706;114.1017	JM
3	1.283	Histidine	C_6_ H_9_ N_3_ O_2_	155.0695	156.0768	0.00	[M + H]^+^	156.0768;110.0711;93.0446;83.0602	JZ
4	1.288	trans-Zeatin	C_10_ H_13_ N_5_ O	219.1120	220.1193	0.11	[M + H]^+^	220.1193;202.1075;148.0618;136.0619	JZ
5	1.410	Glutamic acid	C_5_ H_9_ N O_4_	147.0532	146.0460	0.28	[M-H]^−^	148.0604;130.0499;102.0549;84.0443	JM
6	1.435	Pipecolic acid	C_6_ H_11_ N O_2_	129.0789	130.0862	−0.51	[M + H]^+^	130.0862;84.0807;56.0494	JZ
7	1.476	Glutamine	C_5_ H_10_ N_2_ O_3_	146.0693	145.0620	0.88	[M-H]^−^	145.0621;128.0355;127.0515;109.0409;102.0561;84.046	JZ
8	1.504	Alanin	C_3_ H_7_ N O_2_	89.0476	90.0549	−1.18	[M + H]^+^	90.0549;44.0495	JM
9	1.516	Threonine	C_4_ H_9_ N O_3_	119.0582	118.0511	−0.21	[M + H]^+^	120.0654;102.0548;74.1600;56.0494	JM
10	1.549	Trehalose	C_12_ H_22_ O_11_	342.1161	341.1089	−0.21	[M-H]^−^	341.1090;179.0561;161.046;	JM
11	1.584	Isocorybulbine	C_21_ H_25_ N O_4_	355.1782	356.1858	−0.4	[M + H]^+^	356.1856;192.1019;151.0753	CYHS
12	1.596	Luteolin 7-O-glucoside	C_21_ H_20_ O_11_	448.1007	447.0932	0.23	[M-H]^−^	447.0932;285.0405;133.0296;107.0141	CYH
13	1.596	Quercitrin	C_21_ H_20_ O_11_	448.1006	447.0932	−0.03	[M-H]^−^	447.0933;300.0277;271.0249;255.0301;178.9987;151.00310	CYH
14	1.705	Aspartic Acid	C_4_ H_7_ N O_4_	133.0375	134.0447	0.03	[M-H]^−^	132.0304;116 (115.00376;114.01976);88.0404;74.0246	JZ
15	1.709	Proline	C_5_ H_9_ N O_2_	115.0632	116.0705	−1.04	[M + H]^+^	116.0705;74.0237;70.0651	JM
16	1.895	Valine	C_5_ H_11_ N O_2_	117.0789	118.0862	−0.67	[M + H]^+^	118.0862;72.0808;58.0651;55.0543	JZ, JM
17	1.964	Isoleucine	C_6_ H_13_ N O_2_	131.0946	132.1018	−0.63	[M + H]^+^	132.1017;86.0963	JZ, JM
18	2.021	5-Hydroxytryptophol	C_10_ H_11_ N O_2_	177.0790	178.0862	0.14	[M + H]^+^	178.0865;160.0606;132.0661	JM
19	2.236	Leucine	C_6_ H_13_ N O_2_	131.0946	132.1018	−0.63	[M + H]^+^	132.1018;86.0963;69.0698	JZ
20	2.237	Tyrosine	C_9_ H_11_ N O_3_	181.0739	165.0546	0.21	[M + H]^+^	182.1814;165.0546;147.0443;136.0752;123.0439;119.0491;91.0539	JZ, JM
21	2.243	Pyroglutamic acid	C_5_ H_7_ N O_3_	129.0426	130.0499	0.02	[M + H]^+^	130.0498;84.0443;56.0494	JZ
22	2.370	Phenylalanine	C_9_ H_11_ N O_2_	165.0790	166.0863	0.24	[M + H]^+^	166.0870;120.0809;103.0543;93.0697;91.0542;79.0542	JZ
23	3.067	Tryptophan	C_11_ H_12_ N_2_ O_2_	204.0898	203.0825	−0.54	[M-H]^−^	203.0825;142.0667;116.0506;74.0247	CYHS
24	3.314	4-Methoxysalicylic acid	C_8_ H_8_ O_4_	168.0423	169.0495	−0.03	[M-H]^−^	167.0349;123.0453	JM
25	3.345	Ethylmorphine	C_19_ H_23_ N O_3_	313.1679	314.1752	0.45	[M + H]^+^	314.1751;229;257	CYHS
26	4.113	Chlorogenic acid	C_16_ H_18_ O_9_	354.0950	353.0877	−0.14	[M-H]^−^	353.0884;191.0562;179.0351;161.0244;135.0453	CYH
27	4.820	3,4-Methylenedioxyphenol	C_7_ H_6_ O_3_	138.0317	137.0245	0.11	[M-H]^−^	137.0245;109.0296;107.0141	XX
28	5.435	kakuol	C_10_ H_10_ O_4_	194.0580	195.0652	0.19	[M + H]^+^	195.0651;147.0440	XX
29	5.442	Caffeic acid	C_9_ H_8_ O_4_	180.0423	179.0350	0.12	[M-H]^−^	179.0349;135.0453	CYH
30	5.500	Boldine	C_19_ H_21_ N O_4_	327.1472	328.1545	0.54	[M + H]^+^	328.1548;297.1139;265.0859;178.0864	CYHS
31	5.500	isoboldine	C_19_ H_21_ N O_4_	327.1472	328.1514	0.54	[M + H]^+^	328.1538;297.1120;282.0882;265.0859;253.0841;237.0909;233.0595	CYHS
32	5.784	Yuanhunine	C_21_ H_25_ N O_4_	355.1785	356.1858	0.32	[M + H]^+^	356.1856;192.1019;177.0786	CYHS
33	5.908	Dihydromelilotoside	C_15_ H_20_ O_8_	328.1159	327.1087	0.36	[M-H]^−^	327.1068;147.0452	RG
34	5.909	Suberic acid	C_8_ H_14_ O_4_	174.0892	173.0819	−0.14	[M-H]^−^	173.0819;111.0816;83.0502;53.0345	JM
35	5.952	Protopine	C_20_ H_19_ N O_5_	353.1265	354.1337	0.38	[M + H]^+^	354.1325;336.1221;275.0710;247.0750;206.0812;189.0786;188.0707;149.0598	CYHS
36	6.005	tetrahydropalmatine	C_21_ H_25_ N O_4_	355.1784	356.1856	0.02	[M + H]^+^	356.1859;341.1624;192.1020;165.0912	CYHS
37	6.130	3,4-Dihydroxybenzoic acid	C_7_ H_6_ O_4_	154.0267	153.0194	0.54	[M-H]^−^	153.0194;134.6277;109.0296	RG, CYH
38	6.137	4-Hydroxybenzaldehyde	C_7_ H_6_ O_2_	122.0296	121.0296	0.15	[M-H]^−^	121.0296;92.0268	JZ
39	6.137	Tetrahydrocoptisine	C_19_ H_17_ N O_4_	323.1158	324.1232	0.16	[M + H]^+^	324.1230;176.0705;149.0597	CYHS
40	6.173	Scoulerine	C_19_ H_21_ N O_4_	327.1471	328.1544	0.24	[M + H]^+^	328.1542;178.0862;151.0753;119.0491;91.0542	CYHS
41	6.558	Corybulbine	C_21_ H_25_ N O_4_	355.1784	356.1857	0.22	[M + H]^+^	356.1857;179.1067;178.0861;163.0622	CYHS
42	6.574	4’-Hydroxyacetophenone	C_8_ H_8_ O_2_	136.0524	135.0452	−0.12	[M-H]^−^	135.0450;120.0214	JZ
43	6.582	Dihydroberberine	C_20_ H_19_ N O_4_	337.1314	338.1387	0.03	[M + H]^+^	338.1386;322.1073;320.0922;306.1123;294.1123;293.1047;280.0965	CYHS
44	6.658	Astragalin	C_21_ H_20_ O_11_	448.1004	447.0930	−0.34	[M-H]^−^	447.0931;285.0402;284.0325;256.0368;227.0350	XX, CYH
45	6.690	Allocryptopine	C_21_ H_23_ N O_5_	369.1577	370.1650	0.21	[M + H]^+^	370.1646;352.1549;291.1005;290.0937;188.0705	CYHS
46	6.782	isoquercetin	C_21_ H_20_ O_12_	464.0956	463.0883	0.21	[M-H]^−^	463.0885;301.0355;300.0277;271.0250;255.03001;151.0039	CYH
47	6.914	7-O-Methylluteolin	C_16_ H_12_ O_6_	300.0634	301.0706	−0.09	[M-H]^−^	299.0559;284.0326;256.0385;227.0349;151.0039;133.0300	CYH
48	6.968	Anethol	C_10_ H_12_ O	148.0888	181.1223	−0.26	[M + H]^+^	149.0960;105.0701;91.0541	RG
49	7.004	4-(3-Hydroxybutyl)-2-methoxyphenol	C_11_ H_16_ O_3_	196.1100	197.1172	0.02	[M + H]^+^	197.1173;179.1065;161.0962;137.0591;135.0805;133.1011;107.0855;93.0698	GJ
50	7.205	canadine	C_20_ H_21_ N O_4_	339.1472	340.1545	0.47	[M + H]^+^	340.1546;176.0707	CYHS
51	7.266	Berberine	C_20_H_17_NO_4_	335.1159	336.1231	0.27	[M + H]^+^	336.1228;320.0916;306.0758;278.0810	CYHS
52	7.301	(+)-Corypalmine	C_20_ H_23_ N O_4_	341.1627	342.1701	−0.07	[M + H]^+^	342.1698;178.0860;151.0752;119.0489;91.05410	CYHS
53	7.355	Benzaldehyde	C_7_ H_6_ O	106.0418	107.0490	−1.09	[M + H]^+^	107.0491;79.0542	RG
54	7.518	Ferulic acid	C_10_ H_10_ O_4_	194.0579	193.0505	−0.28	[M + H]^+^	195.0644;177.0546;145.0284;121.0626;117.0334;89.0383	XX
55	7.838	Sinapinic acid	C_11_ H_12_ O_5_	224.0685	207.0652	−0.05	[M-H]^−^	223.0613;208.0373;164.0477;179.0716;149.0244	JZ
56	7.846	Quercetin	C_15_ H_10_ O_7_	302.0426	301.0353	−0.06	[M-H]^−^	301.0352;151.0037;149.0244;107.0128	CYH
57	7.973	Jaranol	C_17_ H_14_ O_6_	314.0791	315.0864	0.28	[M + H]^+^	315.180859;300.0625	CYH
58	8.224	Myristicin	C_11_ H_12_ O_3_	192.0787	193.0859	0.12	[M + H]^+^	193.0855;161.0609	XX
59	8.256	Cryptopine	C_21_ H_23_ N O_5_	369.1577	370.1650	0.23	[M + H]^+^	370.2011;205.1095;190.0862;165.0808;150.0674	CYHS
60	8.277	Cinnamic acid	C_9_ H_8_ O_2_	148.0524	147.0452	−0.15	[M-H]^−^	147.0453;102.9488	RG
61	8.454	Corydaline	C_22_ H_27_ N O_4_	369.1939	370.2012	−0.20	[M + H]^+^	370.2013;205.1098;192.1019;179.1067;165.0910;151.0753;150.0675;136.0517;135.0448	CYHS
62	8.468	Medioresinol	C_21_ H_24_ O_7_	388.1523	387.1449	0.18	[M-H]^−^	387.1443;207.0664;163.0756	CYHS
63	8.535	Sebacic acid	C_10_ H_18_ O_4_	202.1205	201.1132	−0.24	[M-H]^−^	201.1132;186.1027;139.1130	JM
64	8.606	Coumarin	C_9_ H_6_ O_2_	146.0371	147.0445	2.26	[M + H]^+^	147.0441;119.0491;103.0542;91.0542	GJ, JZ
65	9.075	Naringenin	C_15_ H_12_ O_5_	272.0684	273.0758	−0.14	[M-H]^−^	271.0610;151.0037	XX, CYH
66	9.161	Kaempferol	C_15_ H_10_ O_6_	286.0478	285.0405	0.30	[M + H]^+^	287.0549;258.0516;165.0184;133.0282;121.0284	XX, CYH
67	9.178	Syringaldehyd	C_9_ H_10_ O_4_	182.0579	181.0506	−0.15	[M-H]^−^	181.0510;166.0272;151.0038	XX
68	9.184	Gingerenone B	C_22_ H_26_ O_6_	386.173	387.1804	0.26	[M + H]^+^	387.1804;207.1015;193.0863;167.0699;137.0596	GJ
69	9.230	dihydrochelerythrine	C_21_ H_19_ N O_4_	349.1317	350.1389	0.73	[M + H]^+^	350.1384;334.1072;319.1214;290.0822	CYHS
70	9.359	Genkwanin	C_16_ H_12_ O_5_	284.0684	283.0611	−0.30	[M-H]^−^	283.0249;268.0365;211.0400;117.0347	CYH
71	9.657	Umbelliferone	C_9_ H_6_ O_3_	162.0317	163.0390	−0.05	[M + H]^+^	163.0396;135.0803;119.0488;107.0491;91.0542	CYH
72	9.720	Matairesinol	C_20_ H_22_ O_6_	358.1417	357.1343	0.07	[M-H]^−^	357.1345;342.1106;298.9558;221.0819;161.0616;147.0453;16137.0609;122.0373	CYH
73	9.720	Camphoric acid	C_10_ H_16_ O_4_	200.1049	199.0976	0.08	[M-H]^−^	199.0973;155.1078	JM
74	9.748	p-Coumaric acid	C_9_ H_8_ O_3_	164.0474	163.0401	0.30	[M-H]^−^	163.0401;162.0312;162.1440;145.0297;135.0452;93.0347;75.1221;74.9537	RG
75	9.825	Apigenin	C_15_ H_10_ O_5_	270.0527	271.0599	−0.57	[M-H]^−^	269.0459;149.0247;117.034;107.0140	CYH
76	9.936	Cinnamaldehyde	C_9_ H_8_ O	132.0574	133.0646	−1.12	[M + H]^+^	133.0647;115.0541;91.0541;79.0542	RG
77	9.942	Epipinoresinol	C_20_ H_22_ O_6_	358.1417	357.1344	0.20	[M-H]^−^	357.1343;151.0760	XX
78	10.554	Pluviatilol	C_20_ H_20_ O_6_	356.1259	357.1330	−0.27	[M + H]^+^	357.1330;339.1226;205.0858;191.0871	XX
79	10.582	alpha-Asarone	C_12_ H_16_ O_3_	208.1100	207.1026	0.13	[M + H]^+^	209.1167;194.0934;178.099;168.0779;153.0544	XX
80	10.758	Cinnamyl alcohol	C_9_ H_10_ O	134.0731	135.0804	−0.36	[M + H]^+^	135.0804;105.0697;91.0541;79.0541	RG
81	10.872	Bergapten	C_12_ H_8_ O_4_	216.0422	217.0495	−0.34	[M + H]^+^	217.0502;202.0268;173.0600	JM
82	11.021	Sesamolin	C_20_ H_18_ O_7_	370.1053	371.1126	0.06	[M + H]^+^	371.1129;353.0912;203.0862;135.0440	XX
83	11.246	Luteolin	C_15_ H_10_ O_6_	286.0478	285.0405	0.05	[M-H]^−^	285.0405;241.0508;217.0512;151.0039;133.0297	CYH
84	11.274	zingerone	C_11_ H_14_ O_3_	194.0943	195.1016	−0.10	[M + H]^+^	195.1015;161.0027;137.0592;117.0281	GJ
85	11.330	4-Hydroxy-3-methoxycinnamaldehyde	C_10_ H_10_ O_3_	178.0630	179.0703	−0.04	[M + H]^+^	179.1701;161.0599;147.0439;119.0490;91.0542;65.0385	RG
86	11.388	Jasmonic acid	C_12_ H_18_ O_3_	210.1254	211.1327	−0.73	[M-H]^−^	209.1182;165.1284;59.012	JZ
87	11.389	Gingerdiol	C_17_ H_28_ O_4_	296.1988	295.1915	0.18	[M-H]^−^	295.19135;280.1680	GJ
88	11.410	O-Methoxycinnamaldehyde	C_10_ H_10_ O_2_	162.0681	163.0754	−0.03	[M + H]^+^	163.0754;133.0650;131.0492;107.0491;105.0698;91.0542;79.0541	RG
89	11.703	Citral	C_10_ H_16_ O	152.1201	153.1274	0.18	[M + H]^+^	153.1272;135.1168;95.08855;71.0491;69.0697;59.0491	JM
90	11.864	Velutin	C_17_ H_14_ O_6_	314.0790	315.0862	−0.28	[M + H]^+^	315.0863;300.0629;272.0679	CYH
91	12.008	Methyleugenol	C_11_ H_14_ O_2_	178.0993	179.1066	−0.25	[M + H]^+^	179.1065;164.0828;151.0753;138.0674	XX
92	12.231	Aristololactam	C_17_ H_11_ N O_4_	293.0690	294.0762	0.54	[M + H]^+^	294.0761;279.0527;251.0579;239.0708	XX
93	12.370	10-Hydroxydecanoic acid	C_10_ H_20_ O_3_	188.1412	187.1340	0.00	[M-H]^−^	187.1340;141.1286	JM
94	12.488	Hexadecanedioic acid	C_16_ H_30_ O_4_	286.2145	285.2072	0.15	[M-H]^−^	285.2072;267.1966;241.2170	JM
95	13.218	Azelaic acid	C_9_ H_16_ O_4_	188.1048	187.0975	−0.10	[M-H]^−^	187.0975;143.1075;125.0972;123.0811;97.0659	JM
96	13.321	Andrographolide	C_20_ H_30_ O_5_	350.2094	351.2167	0.28	[M-H]^−^	349.2021;331.1905;287.2016	JM
97	13.426	Gingerenone A	C_21_ H_24_ O_5_	356.1625	357.1699	0.39	[M + H]^+^	357.1709;339.1235;177.091;163.075;137.0596;145.0649;131.0490	GJ
98	13.668	palmatine	C_21_ H_22_ N O_4_	352.1550	353.1623	0.35	[M + H]^+^	353.1624	CYHS
99	13.789	(8)-Gingerol	C_19_ H_30_ O_4_	322.2144	321.2071	−0.19	[M-H]^−^	321.2072;305.0950;127.1130;57.0346	GJ
100	13.809	alpha-Curcumene	C_15_ H_22_	202.1721	203.1794	−0.12	[M + H]^+^	203.1794;161.1326;147.1168;133.1011;119.0853;105.0698;95.0854;81.0698;69.0698	GJ, XX
101	13.935	Aurantiamide	C_25_ H_26_ N_2_ O_3_	402.1944	403.2018	0.09	[M + H]^+^	403.1181;152.1071	CYH
102	13.947	Paradol	C_17_ H_26_ O_3_	278.1882	279.1955	−0.07	[M + H]^+^	279.1952;261.1868;229.1567;177.0912;163.0754;137.1597;131.0490;103.0540;81.0698	GJ
103	14.104	pinolenic acid	C_18_ H_30_ O_2_	278.2244	279.2317	−0.50	[M + H]^+^	279.2318;95.0855;91.0699;67.0542	JM
104	14.642	Gingerdione	C_17_ H_24_ O_4_	292.1677	275.1643	0.67	[M + H]^+^	293.1749;233.1537;163.0755;137.0597;131.0492	GJ
105	14.657	(8)-Gingerdione	C_19_ H_28_ O_4_	320.1988	321.2061	0.21	[M + H]^+^	321.2061;303.2339;261.1852;229.1589;207.1375;191.1075;163.0753;137.0596;95.0853	GJ
106	14.778	[6]-Dehydrogingerdione	C_17_ H_22_ O_4_	290.1518	291.1591	0.05	[M + H]^+^	291.1590;270.4919;177.0545;145.0283;103.2844;89.0384;71.0854	GJ
107	14.905	(10)-Shogaol	C_21_ H_32_ O_3_	332.2352	333.2425	0.29	[M + H]^+^	333.3425;315.2318;197.1911;177.0909;162.0673;145.0647;137.0596;83.0852	GJ
108	15.224	Shogaol	C_17_ H_24_ O_3_	276.1727	277.1799	0.37	[M + H]^+^	277.1810;137.0597	GJ
109	15.225	Safrole	C_10_ H_10_ O_2_	162.0681	163.0753	−0.17	[M + H]^+^	163.0753;148.0519;135.0803;133.0648;131.0492;121.0647;120.0568;115.0541;107.0499;105.1697;103.0541;91.0544;79.0541;77.0386;55.0178	XX
110	15.284	(10)-Gingerol	C_21_ H_34_ O_4_	350.2457	349.2384	−0.10	[M + H]^+^	351.2510;333.2384;315.2331;179.0603;137.0595;83.0856	GJ
111	15.507	6-Gingerol	C_17_ H_26_ O_4_	294.1832	277.1799	0.22	[M-H]^−^	293.1763;236.1053;221.1545;177.0927	GJ
112	15.822	Benzoic acid	C_7_ H_6_ O_2_	122.0369	121.0297	1.30	[M-H]^−^	121.0294;77.040	CYH
113	16.210	Estriol	C_18_ H_24_ O_3_	288.1726	321.2062	0.18	[M-H]^−^	287.2230;171.1028;145.0870;143.1080	XX
114	16.245	(+)-syringaresinol	C_22_ H_26_ O_8_	418.1627	417.1554	−0.24	[M-H]^−^	417.1550;387.1114;181.0507;166.0260	CYH
115	16.563	16-Hydroxyhexadecanoic acid	C_16_ H_32_ O_3_	272.2352	271.2279	0.02	[M-H]^−^	271.2278;253.2187;225.2222;155.1440	JM
116	16.634	(8)-Shogaol	C_19_ H_28_ O_3_	304.2039	305.2112	0.09	[M + H]^+^	305.2112;287.2013;177.0908;162.0672;137.0596;95.0856;83.0855	GJ
117	16.862	12-Gingerol	C_23_ H_38_ O_4_	378.2771	377.2698	0.31	[M + H]^+^	379.284;361.273;343.254;177.1632;137.0961;109.1014;95.0854	GJ
118	17.139	Linolenic Acid	C_18_ H_30_ O_2_	278.2246	279.2319	−0.05	[M + H]^+^	279.2316;179.1436;125.0963	JM
119	17.472	10-Gingerdione	C_21_ H_32_ O_4_	348.2302	349.2375	0.39	[M + H]^+^	349.2286;331.2265;193.0867;177.0901;155.1429;145.0283;135.0451;83.0857;57.0698	GJ
120	17.512	myristic acid	C_14_ H_28_ O_2_	228.2090	227.2017	0.34	[M-H]^−^	227.2016	XX
121	17.625	Methyl-6-Gingerol	C_18_ H_28_ O_4_	308.1989	309.2061	0.35	[M + H]^+^	309.2008;291.1955;276.9002;177.1279;151.1118;99.0804;71.0855	GJ
122	18.510	Linoleic acid	C_18_ H_32_ O_2_	280.2402	279.1229	−0.12	[M-H]^−^	279.2326;261.2224	XX, JM, CYH
123	18.937	Ricinoleic Acid	C_18_ H_34_ O_3_	298.2507	297.2435	−0.21	[M-H]^−^	297.2436;279.2333;183.1387	JM
124	20.326	(4)-Gingerol	C_15_ H_22_ O_4_	266.1553	265.1480	12.98	[M-H]^−^	267.1595;189.1285;169.0865;151.0751;119.0855;83.0489	GJ
125	21.601	Stearic acid	C_18_ H_36_ O_2_	284.2716	283.2643	0.25	[M-H]^−^	283.2641;143.5420	RG
126	21.635	Oleamide	C_18_ H_35_ N O	281.2719	282.2790	0.25	[M + H]^+^	282.2799;265.2522;97.1011;83.0855	JM
127	23.091	Palmitic Acid	C_16_ H_32_ O_2_	256.2403	255.2330	0.23	[M-H]^−^	255.2330;183.0441;116.5201	XX, CYH
128	24.794	Oleic acid	C_18_ H_34_ O_2_	282.2560	281.2486	0.15	[M-H]^−^	281.2485;183.7570	JM, CYH
129	26.875	behenic acid	C_22_ H_44_ O_2_	340.3340	339.3267	−0.39	[M-H]^−^	339.3274;321.3149;183.0122;59.0398	RG

#### 3.1.1 Analysis of the fragmentation pattern of flavonoids components

**Figure 3:**
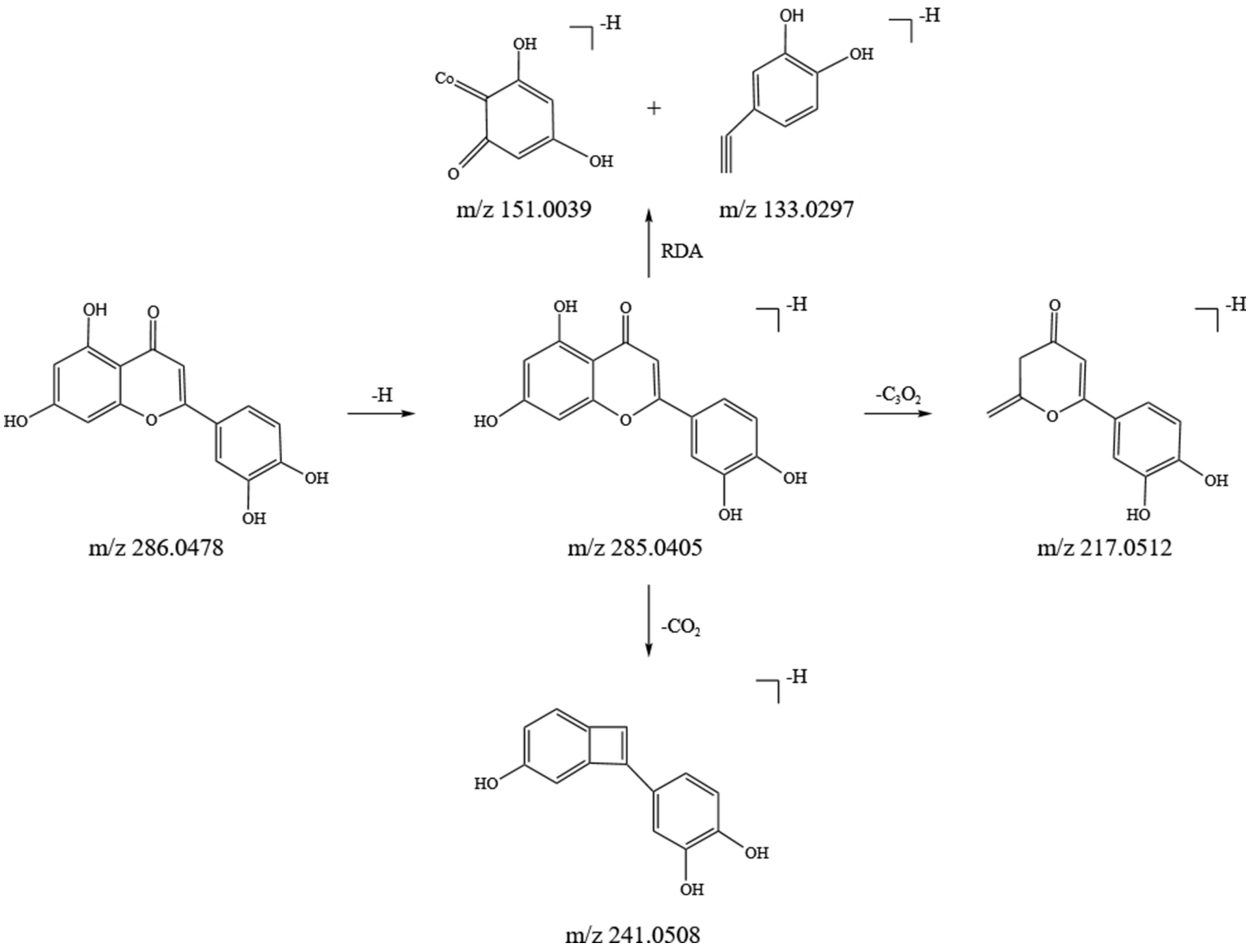
Inference of fragmentation pathway of luteolin quality spectrum.

Flavonoids are key active ingredients in SFTOT. Using UHPLC-Q-Orbitrap/MS, we identified 11 flavonoids in the SFTOT transdermal receiving solution, including: 7-O-Methylluteolin, Genkwanin, Jaranol, Luteolin 7-O-glucoside, Luteolin, Quercetin, Apigenin, Quercitrin, Isoquercetin, Syringaldehyde, Dihydromelilotoside, Naringenin, Astragalin, and Kaempferol. These flavonoids are primarily derived from CYH. The primary fragmentation modes of flavonoids under high-energy collision in mass spectrometry are RDA fragmentation and the loss of neutral ions, such as CO_2_. Taking Luteolin, found in CYH, as an example, we speculated its fragmentation pattern. The excimer ion peak of the compound appeared at m/z 285.0405 [M-H]^−^. The parent ion undergoes RDA cleavage, producing the fragment ions at m/z 151.0039 and m/z 133.0297. The A-ring loses C_3_O_2_, generating the characteristic ion fragment at m/z 217.0512 [M-H-C3O2]^−^, while the C-ring loses CO and O, resulting in the characteristic ion fragment at m/z 241.0508. Based on the ion fragment data and literature (23), we identified the compound as Luteolin, and its fragment ions and potential cleavage pathways are shown in Figure 3.

#### 3.1.2 Analysis of the fragmentation pattern of alkaloid components

**Figure 4:**
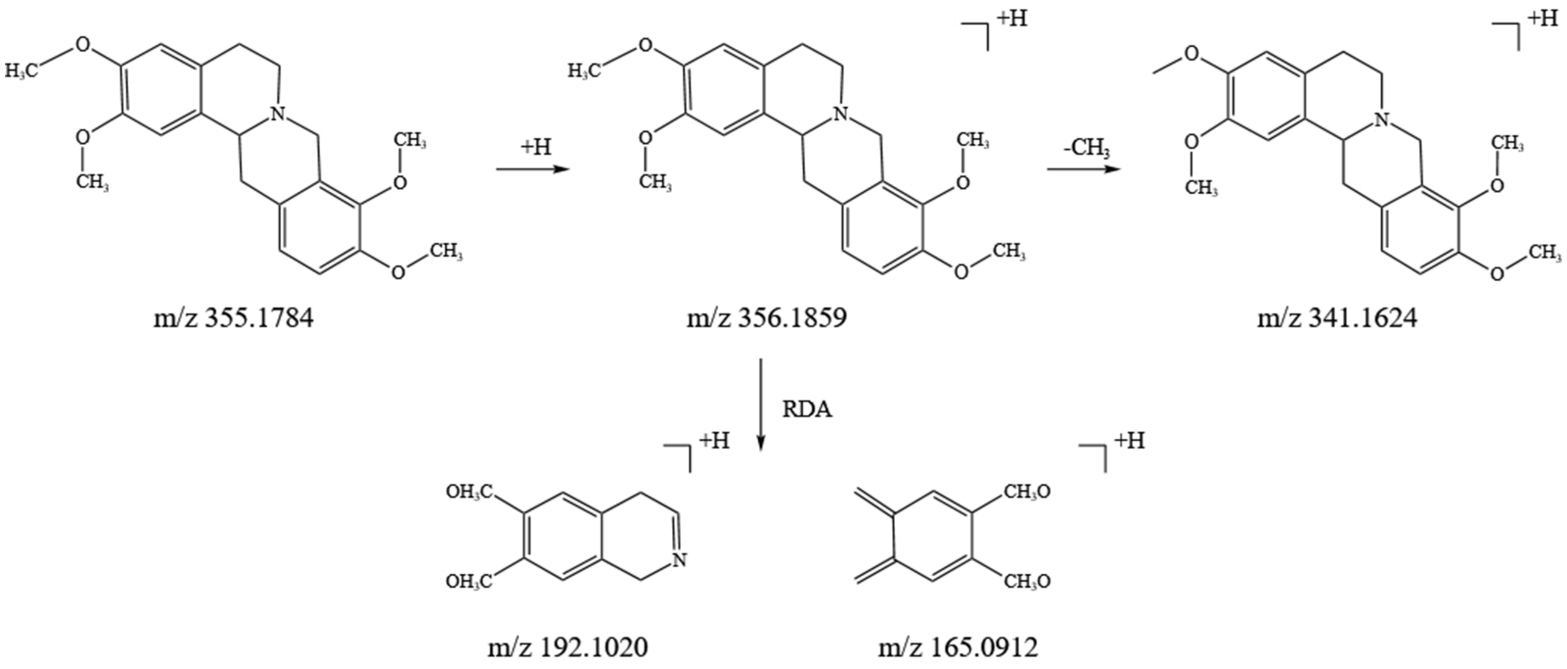
Inference of fragmentation pathway of tetrahydropalmatine quality spectrum.

Alkaloids are a crucial class of natural compounds with diverse biological activities, including anti-inflammatory, analgesic, antioxidant, and nervous system-regulating properties. In this study, we identified 21 alkaloids in the SFTOT transdermal receiving solution, primarily derived from CYHS. Under high-energy collision in mass spectrometry, alkaloids typically undergo RDA fragmentation and the loss of CH₃, among other pathways. Using tetrahydropalmatine, found in CYHS, as an example, we analyzed its fragmentation pattern. The quasi-molecular ion peak appeared at m/z 356.1859 [M + H]^+^. The loss of a CH₃ group generated a fragment at m/z 341.1624 [M + H-CH₃]^+^. The parent nucleus then underwent RDA cleavage, producing complementary fragment ions at m/z 192.1020 and m/z 165.0912. Based on ion fragment data and literature reports (24), we identified the compound as tetrahydropalmatine. Its fragment ions and potential cleavage pathway are illustrated in Figure 4.

#### 3.1.3 Analysis of the fragmentation pattern of gingerol components

**Figure 5:**
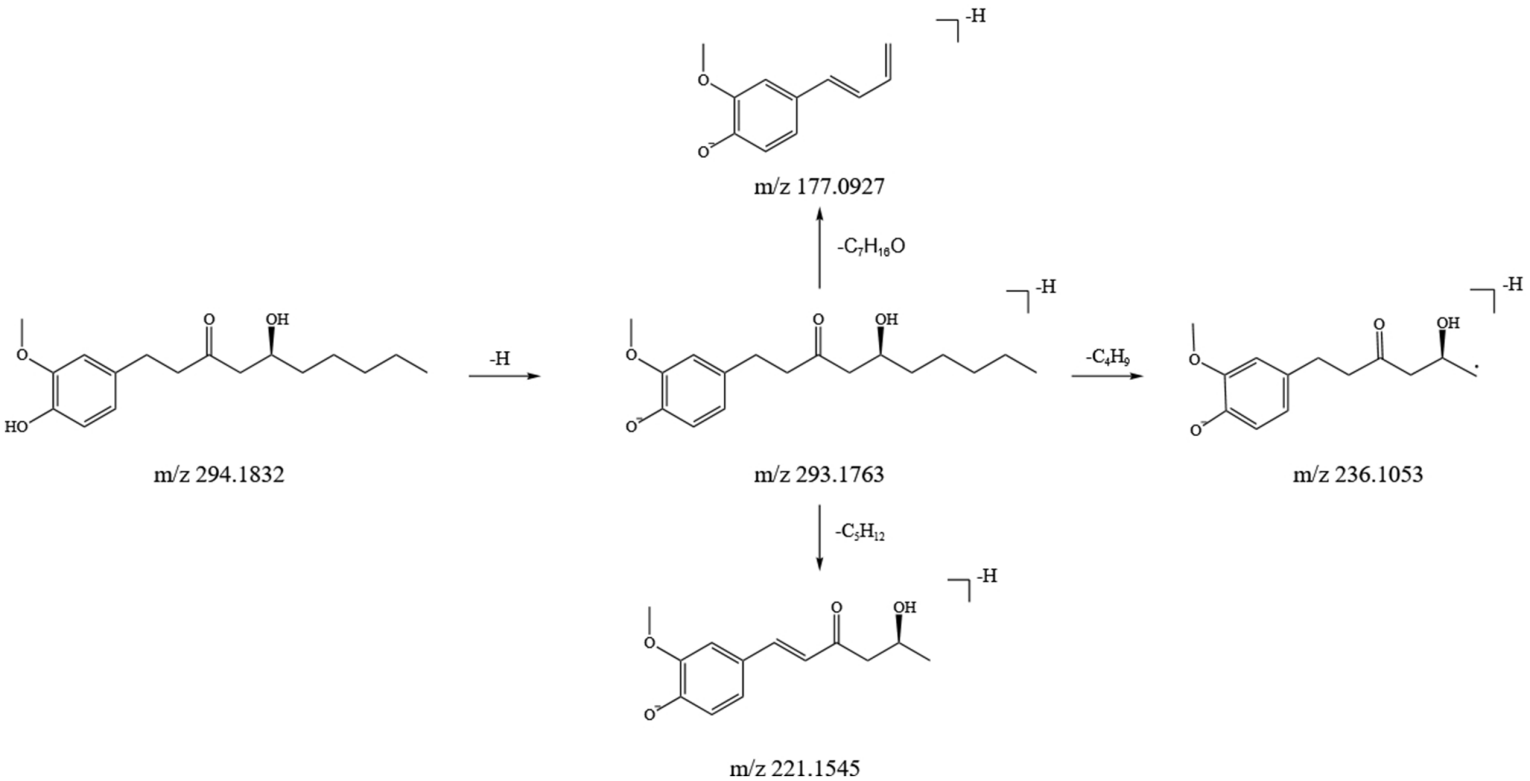
Inference of fragmentation pathway of 6-gingerol quality spectrum.

In this study, we identified 11 gingerol compounds in the SFTOT transdermal receiving solution, all derived from dried ginger. These compounds are characteristic constituents of GJ, sharing a 3-methoxy-4-hydroxyphenyl or a structurally similar parent nucleus. Gingerols exhibit a stronger response in positive ion mode. Under high-energy collision in mass spectrometry, gingerols primarily fragment through the loss of hydroxyl groups from the benzene ring, forming corresponding fragment ions. The side chain cleavage generates characteristic fragments, including m/z 177 (C₁₀H₁₃O₂), m/z 137 (C₈H₉O₂), and small neutral molecules such as CH₃(CH₂)_n_CHO and CH₃COOH, which are readily eliminated. Using 6-Gingerol as an example, we analyzed its fragmentation pattern. The quasi-molecular ion peak appeared at m/z 293.1763 [M-H]^−^. Under high-energy collisions, the main secondary fragment ions included m/z 236.1053, m/z 221.1545, and m/z 177.0927, corresponding to [M-H-C₄H₉]^−^, [M-H-C₅H₁₂]^−^, and [M-H-C₇H₁₆O]^−^, respectively. Based on ion fragment data and literature reports (25), we identified the compound as 6-Gingerol. Its fragment ions and potential cleavage pathway are illustrated in Figure 5.

### 3.2 Network pharmacology study based on Shufeitie ointment transdermal components

#### 3.2.1 Construction of the component-disease database of potential targets

**Figure 6:**
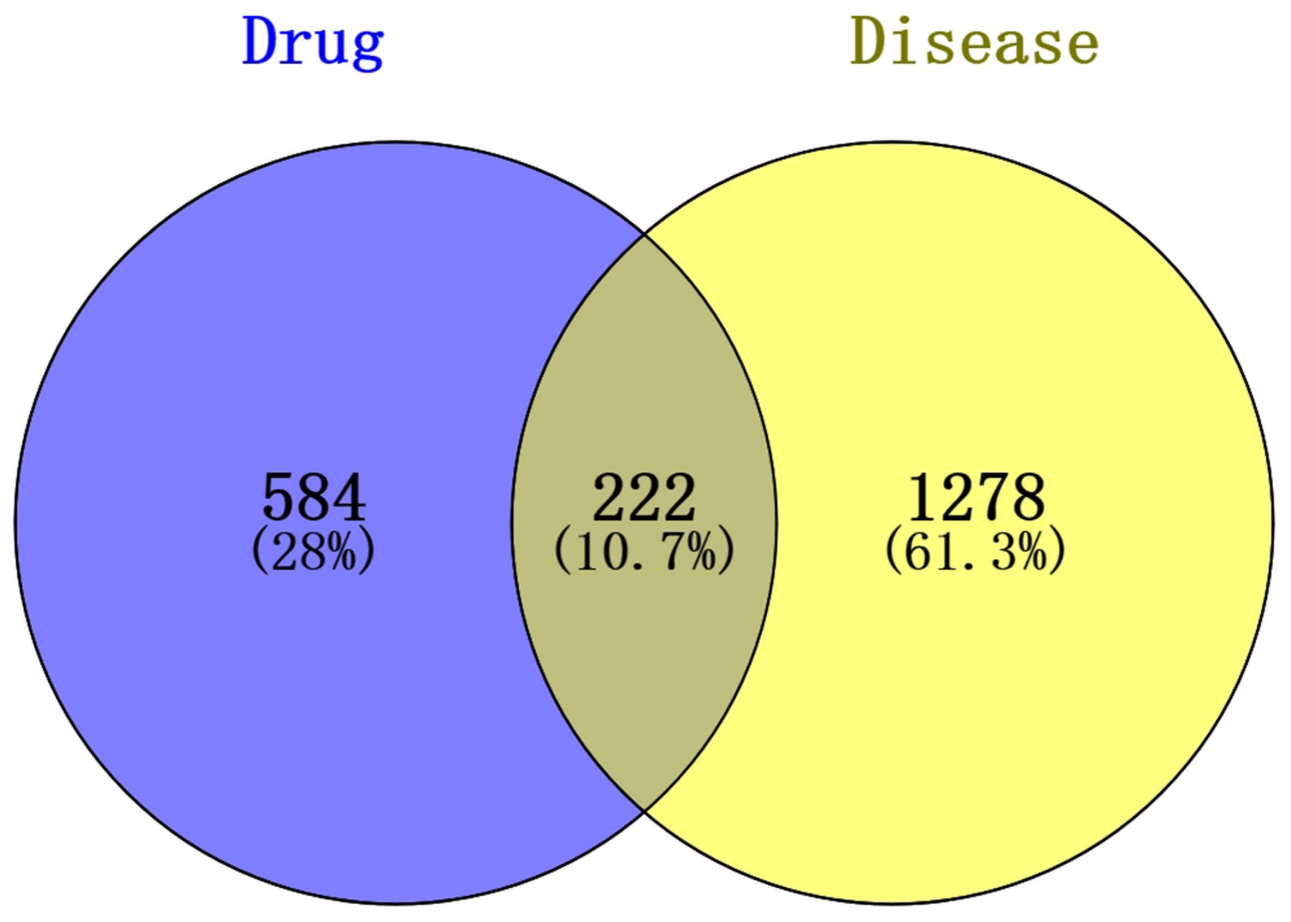
Venn plot of the intersection between SFTOT and COPD targets.

To further explore the potential gene targets of SFTOT in Chronic Obstructive Pulmonary Disease (COPD), we used the SwissTargetPrediction, PharmMapper, and SEA databases to predict targets for the 129 transdermal compounds identified through LC–MS analysis. After removing duplicates and performing UniProt correction, we obtained 806 active ingredient targets. Simultaneously, we searched for COPD-related disease targets in the GeneCards, DrugBank, and OMIM databases using COPD as the keyword. After eliminating duplicates and applying UniProt correction, we identified 1,500 human genes associated with COPD. We then intersected the active ingredient targets with the COPD disease targets, resulting in 222 intersection targets. These targets form the SFTOT target database for the treatment of COPD, and a Venn diagram illustrating the intersection is shown in Figure 6.

#### 3.2.2 Protein–protein interaction network analysis of potential active ingredients

**Figure 7:**
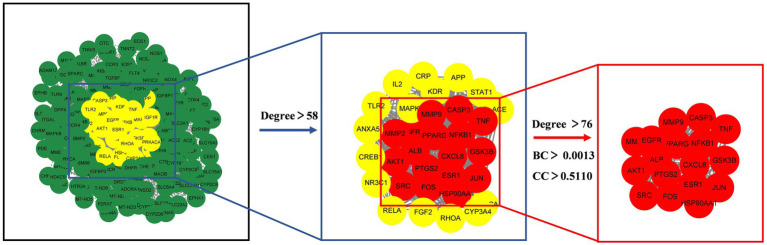
Core target screening process diagram.

The 222 intersection targets were imported into STRING, and a PPI network was constructed using Cytoscape to explore the relationships between the targets. The PPI network revealed a total of 3,927 edges, indicating 3,927 interaction relationships between the proteins. In the network, the Degree value of a node represents the number of connections it has. By examining the Degree value, we identified proteins or molecules that are highly involved in these interactions. We used the CytoNCA plug-in to calculate the network’s topology features and performed two rounds of screening to identify the core targets. The first round used a Degree > 2 times the median, and the second round incorporated Degree, BC, and CC > median. As a result, we identified 17 potential core targets, and the screening process is shown in Figure 7.

**Figure 8:**
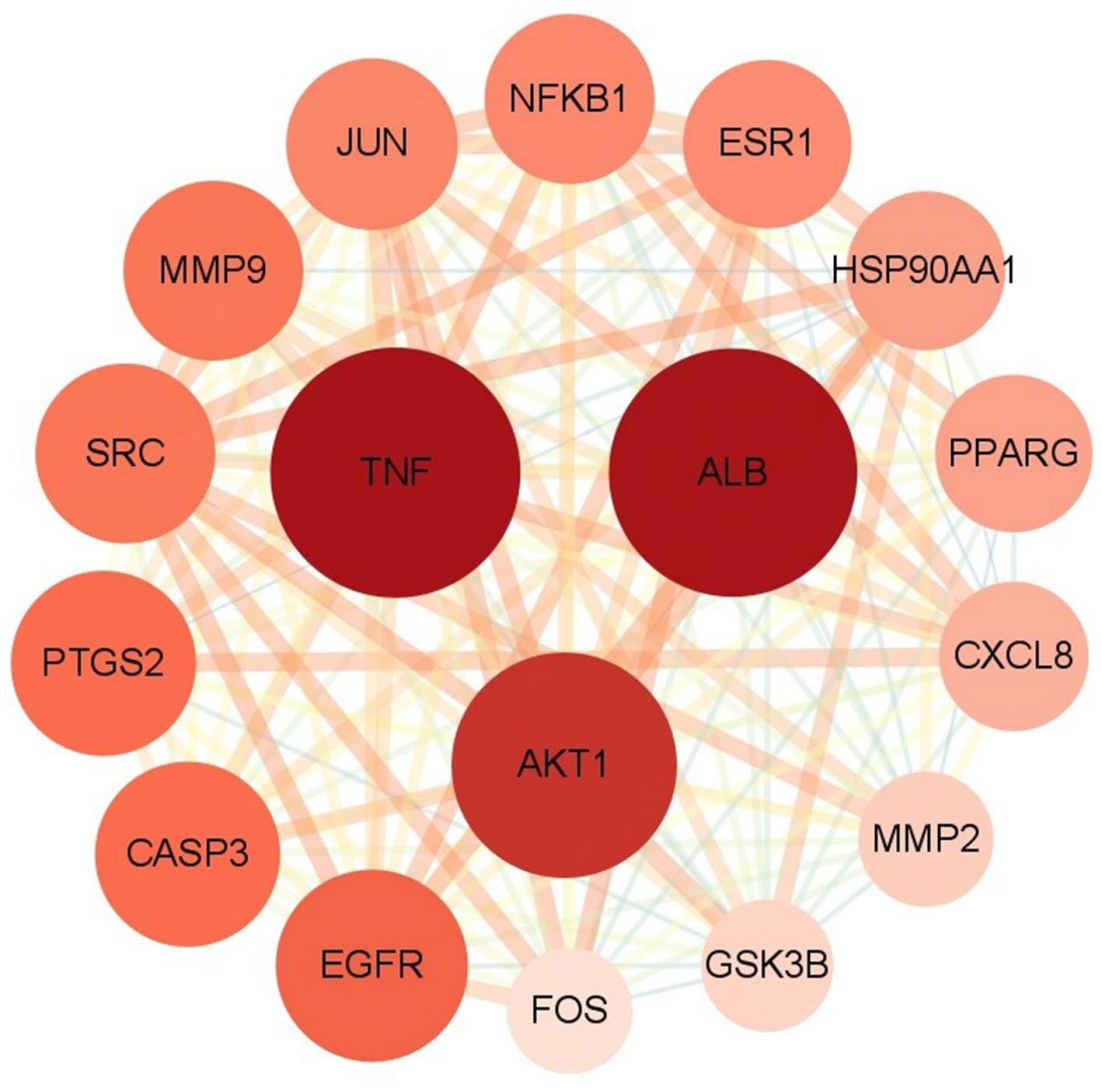
The core targets analyzed by PPI network.

The potential core targets of SFTOT are visually represented in the network, with color and size variations reflecting their degree value. Deeper node colors and larger node sizes indicate higher interaction, while lighter colors and smaller sizes suggest fewer interactions (Figure 8). We focused on the top five targets in the PPI core target network: Tumor necrosis factor (TNF), Albumin (ALB), AKT Serine/Threonine Kinase 1 (AKT1), Epidermal Growth Factor Receptor (EGFR), and Caspase-3 (CASP3). These targets exhibited significant connectivity with other proteins and may represent key targets for the candidate compounds in COPD treatment. Therefore, we selected these five targets for molecular docking verification.

#### 3.2.3 GO and KEGG signaling pathway enrichment analysis

**Figure 9:**
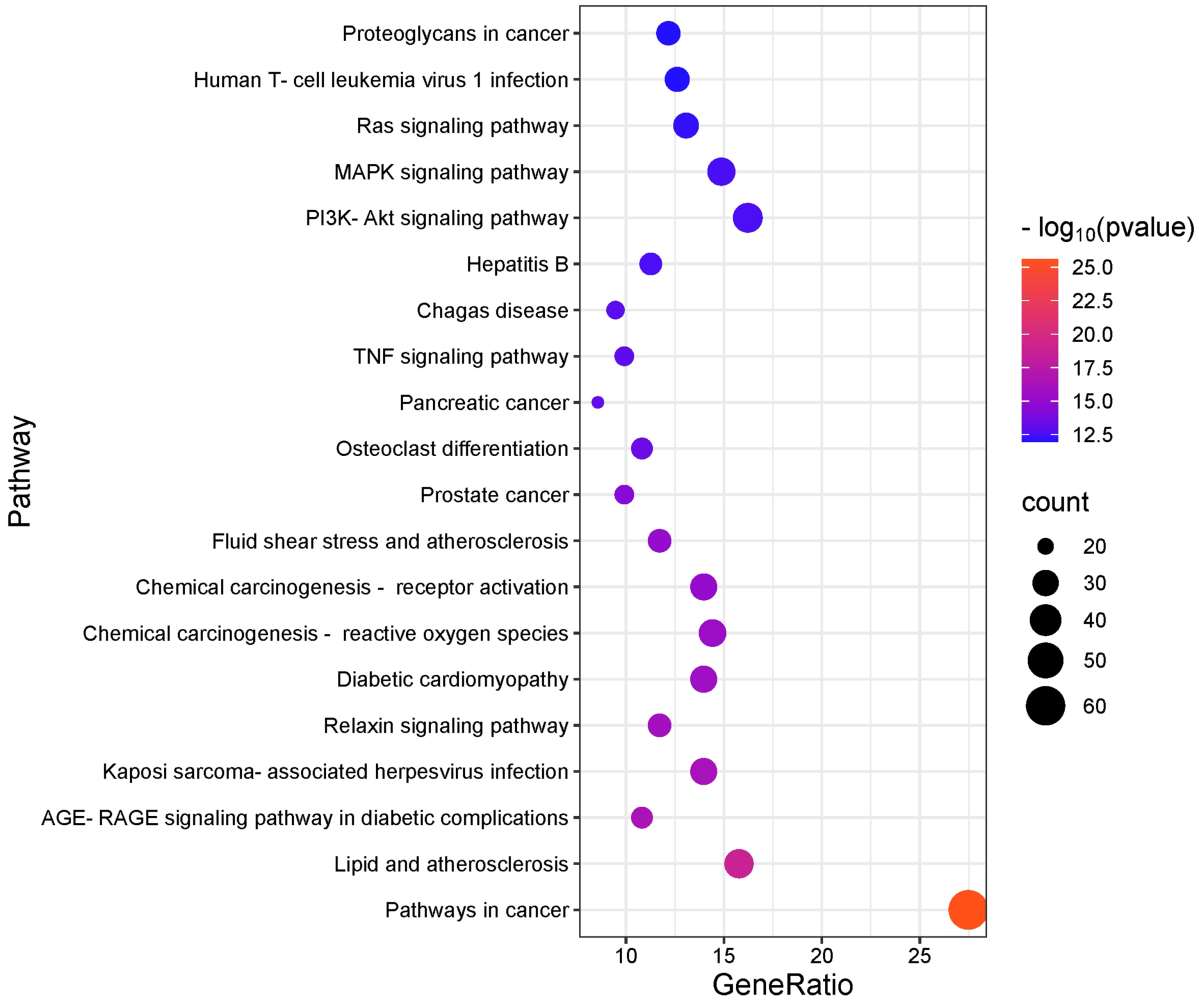
KEGG enrichment analysis of skinpermeable components of SFTOT against COPD (top 20 were listed).

**Figure 10:**
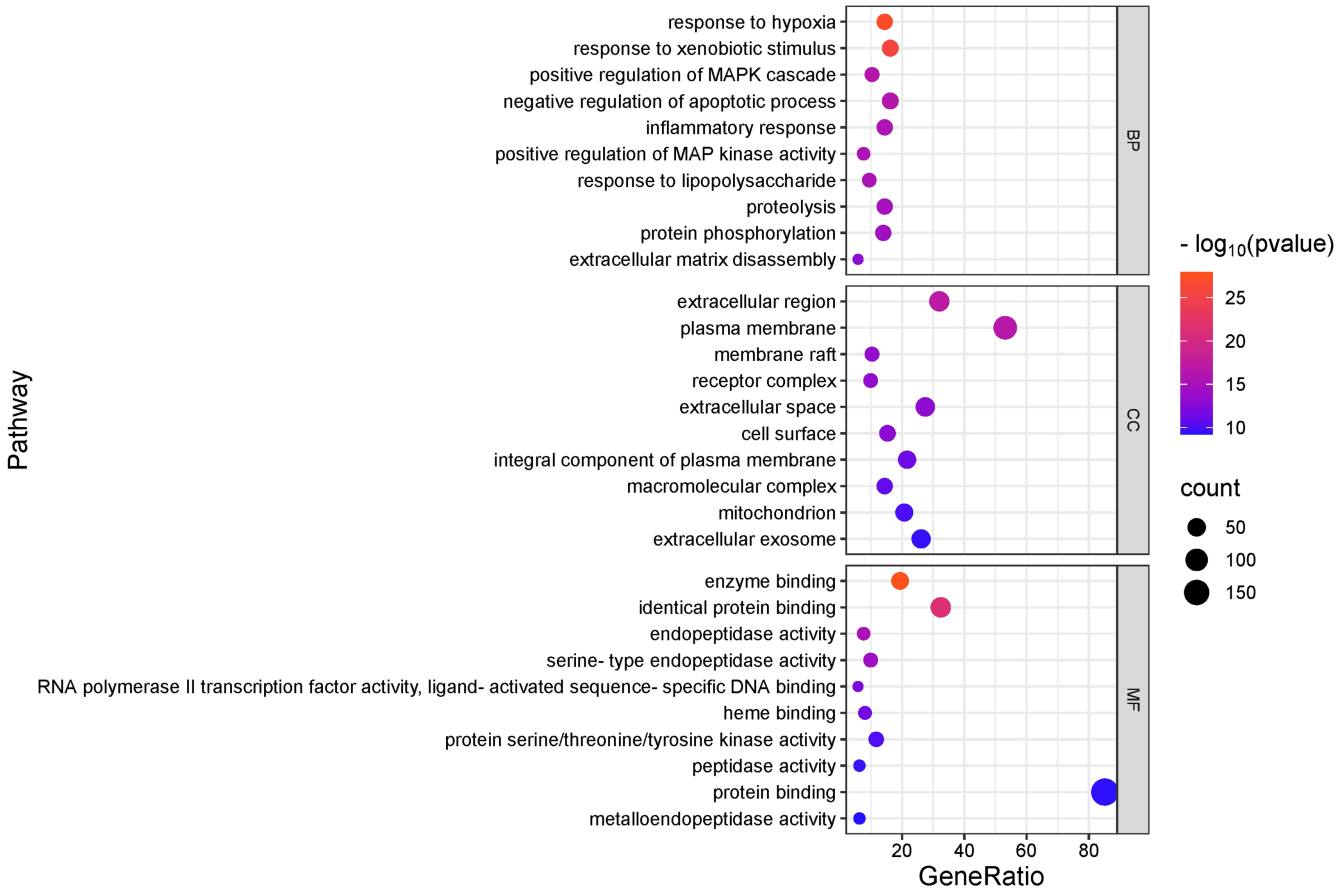
GO enrichment analysis of skinpermeable components of SFTOT against COPD (top 10 were listed). BP, biological process; CC, cellular component; MF, molecular function.

We conducted GO and KEGG analyses of the intersection targets using the DAVID database to identify the molecular functions and signaling pathways regulated by SFTOT. The GO enrichment analysis identified 838 Biological process (BP), 113 cellular component (CC), and 195 Molecular function (MF) with significant relevance. The KEGG pathway analysis revealed 187 significant pathways. The top 20 pathways from KEGG and the top 10 from GO enrichment are presented in bubble plots (Figures 9, 10). In these plots, the ordinate represents the enriched terms, while the abscissa shows the gene ratio for each term. The size of the bubble corresponds to the number of enriched genes, and the color intensity reflects the significance of the enrichment.

Through GO functional enrichment analysis and KEGG analyses, we predicted that SFTOT regulates several key biological components. These include BP such as response to hypoxia, inflammatory response, and the positive regulation of MAP kinase activity and protein phosphorylation. CC include membrane rafts, receptor complexes, and extracellular exosomes; along with endopeptidase activity, protein serine/threonine/tyrosine kinase activity, protein binding and other MF. Furthermore, SFTOT appears to regulate important signaling pathways such as the Advanced Glycation End-products - Receptor for Advanced Glycation End-products (AGE-RAGE), TNF, PI3K/protein kinase B (Akt) signal transduction pathway (PI3K-Akt), and mitogen-activated protein kinase (MAPK) pathways, which may contribute to its therapeutic effects in COPD.

#### 3.2.4 Component-target-pathway network model

**Figure 11:**
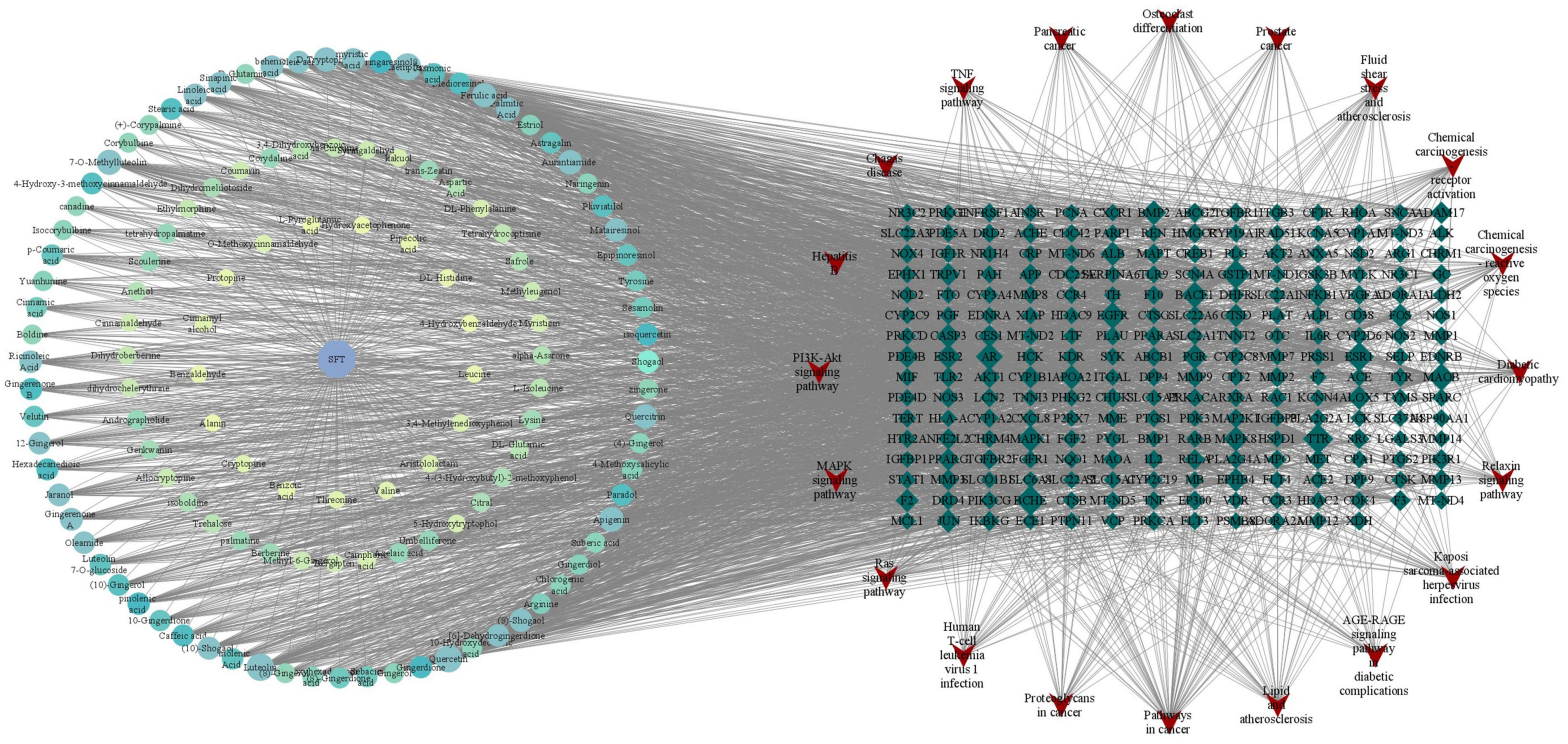
“Component-target-pathway” network model (The hexagon is the component, the blue diamond is the target, and the red inverted triangle is the pathway).

We used Cytoscape 3.7.1 software to construct and analyze the relationship network between the potential active components and targets of SFTOT, resulting in a network diagram with 377 nodes and 3,083 edges. In the diagram, nodes of different colors represent the active components from different drugs (Figure 11). Based on the Degree value and relevant literature, we identified the following as core active ingredients: luteolin, kaempferol, quercetin, 7-O-methylluteolin, apigenin, ferulic acid, palmitic acid, sinapic acid, shogaol, and myristic acid. Among these, Luteolin, quercetin, hydroxygenkwanin, and apigenin are derived from CuYuanHua (CYH). Ferulic acid and myristic acid are from XiXin (XX), while kaempferol and palmitic acid are common to both XX and CYH. Sinapic acid is found in JieZi (JZ), and 6-shogaol originates from GanJiang (GJ). These findings suggest that the core Chinese medicines contributing to SFTOT’s therapeutic effects in this study are CYH, XX, JZ, and GJ. Furthermore, these 10 components play a crucial role in the treatment of COPD.

### 3.3 Molecular docking based on network pharmacology

**Figure 12:**
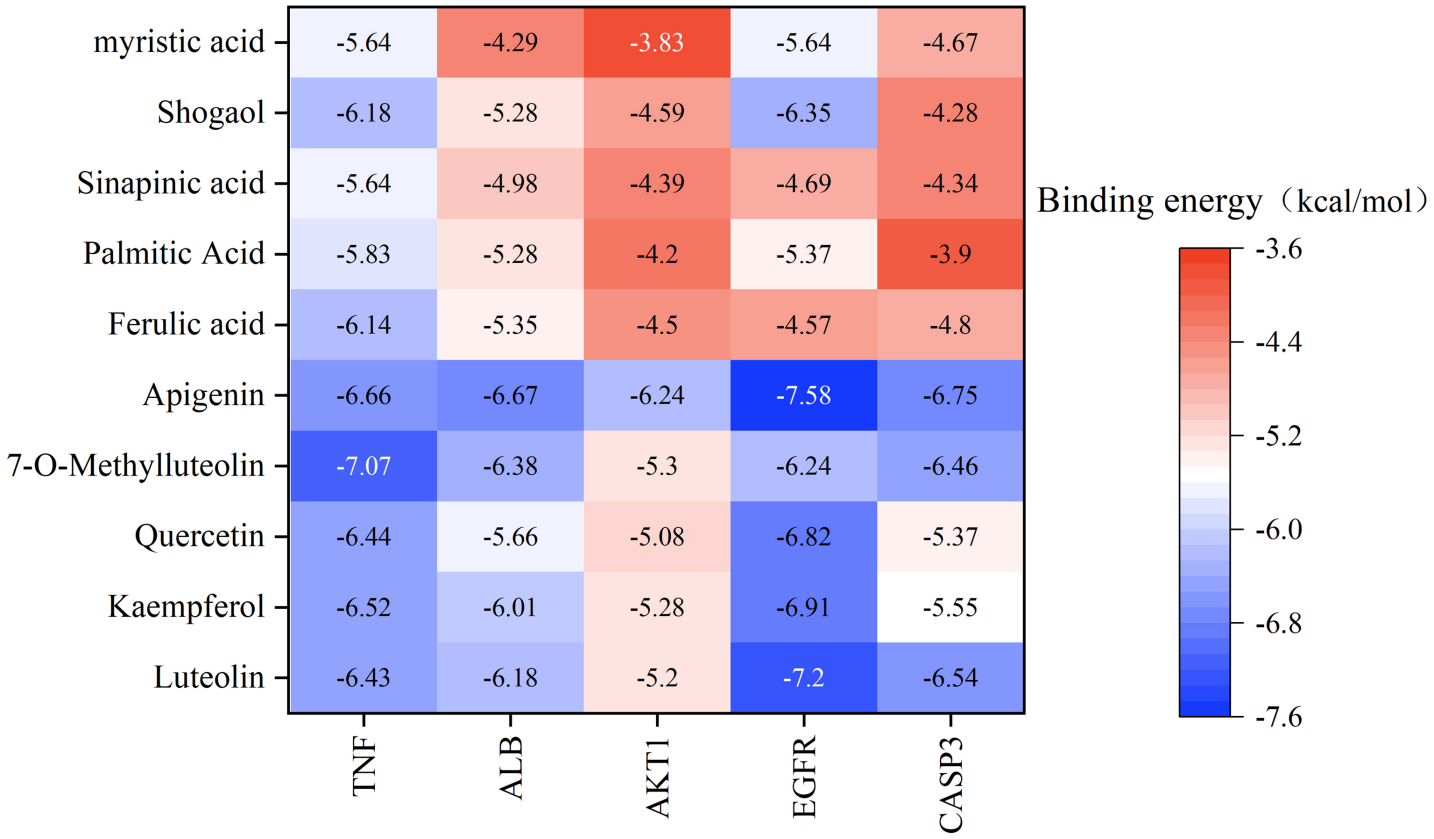
Molecular docking binding energy heat map of targets and active ingredients.

**Figure 13:**
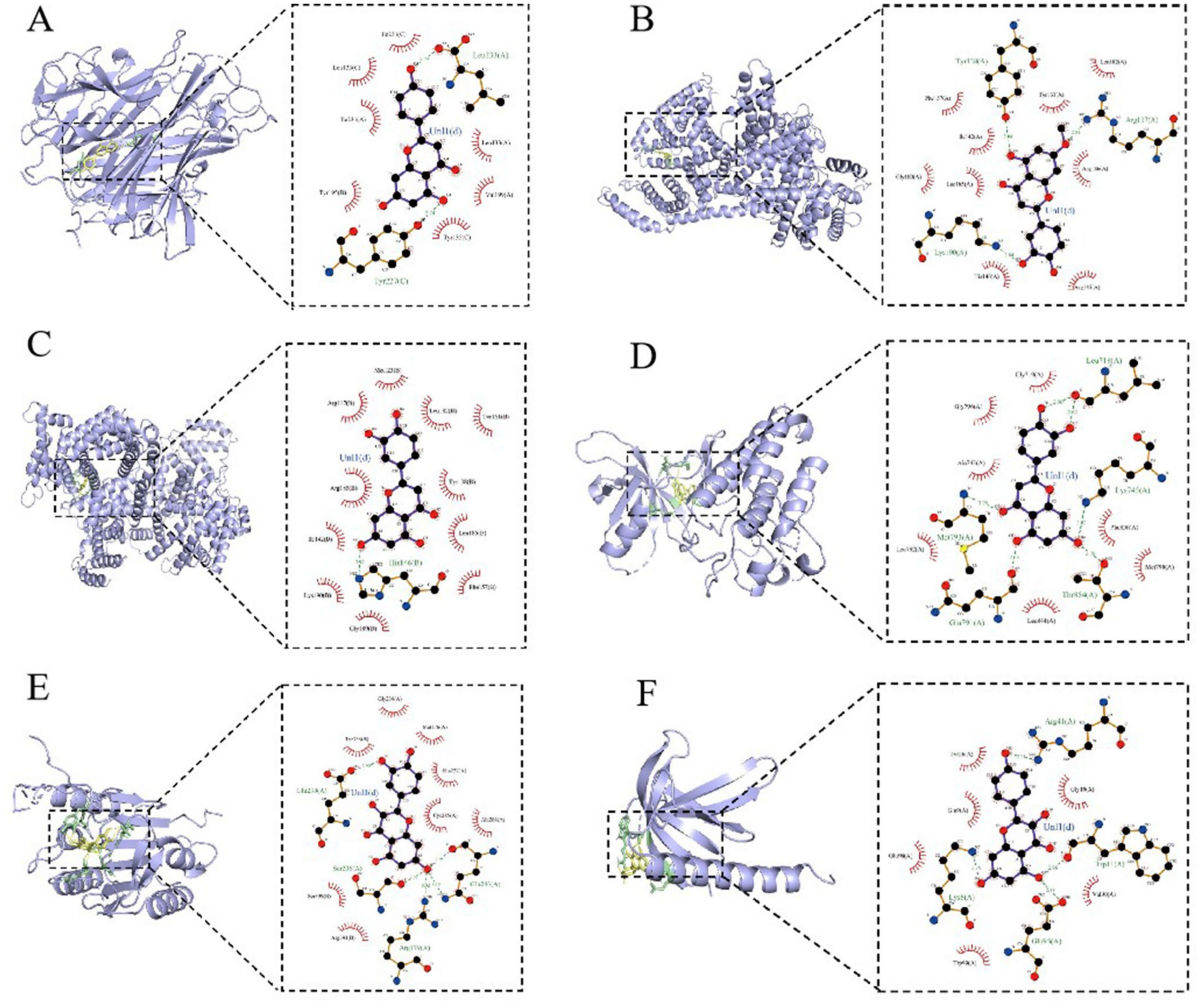
SFTOT core components are interconnected with molecules of core targets. **(A)** Apigenin and TNF. **(B)** 7-O-Methylluteolin and ALB. **(C)** Luteolin and ALB. **(D)** Luteolin and EGFR. **(E)** Quercetin and CASP3. **(F)** Kaempferol and AKT1.

Based on the previous data, we identified the intersection targets and main active components of Shufeitie ointment (SFTOT) in the treatment of Chronic Obstructive Pulmonary Disease (COPD). To explore the key signaling molecules involved, we conducted molecular docking to investigate the interaction between the active components and core targets. AutoDock software was employed for pairwise molecular docking between the 10 selected active components and the five top core targets. The binding free energy was used to evaluate the affinity between the active ingredient (ligand) and the core target receptor. A binding energy of <0 indicates that the ligand and receptor can bind spontaneously. When the binding energy is < −5.0 kcal/mol, it suggests that the ligand and receptor are well docked (26), while a docking energy < −7 kcal/mol indicates a strong binding effect. The docking results are shown in Figure 12. The results with lower binding energy were visualized to show the receptor-ligand complex model. Figure 13 highlights the optimal binding site of the ligand small molecules with the receptor protein. Among the 50 receptor-ligand docking groups, 36 exhibited binding energies < −5.0 kcal/mol, accounting for 72%. This indicates that the core components demonstrated strong binding activity with the core targets. The highest docking score was observed for the interaction between Epidermal growth factor receptor (EGFR) and apigenin, with a binding energy of −7.58 kcal/mol. Apigenin, derived from CuYuanHua (CYH), also exhibited stronger binding with the other core targets compared to the other components. These findings suggest that apigenin is a key component in the therapeutic action of SFTOT. These docking results provide valuable data for further optimization of the SFTOT prescription and dosage forms in future research.

## 4 Discussion

Chronic Obstructive Pulmonary Disease (COPD) is primarily characterized by persistent inflammation of the airways, pulmonary vessels, and lung parenchyma. Its pathogenesis is associated with an imbalance between oxidative stress and antioxidant defenses (2). Although previous studies have demonstrated the significant efficacy of Shufeitie ointment (SFTOT) in treating COPD, its pharmacodynamic components and specific mechanisms of action remain insufficiently understood. Therefore, this study aims to explore the pharmacodynamic basis and mechanisms through which SFTOT exerts its therapeutic effects in COPD, thereby enhancing our understanding of its clinical benefits.

In this study, UHPLC-Q-Orbitrap/MS technology was used to identify the transdermal permeation components of SFTOT. Firstly, the chromatographic conditions were investigated. Referring to the liquid chromatography conditions, combined with the differences of high performance liquid chromatography columns and the characteristics of mass spectrometry, 0.1% formic acid water (A)-acetonitrile (B) was selected as the mobile phase, the column temperature was 40°C, and the flow rate was 0.2 mL/min. The HPLC chromatographic gradient was tried first, and the time was shortened on the basis of the original gradient. It was found that the samples were completely eluted before 18 min and the peak time was compact, concentrated between 12–18 min. Therefore, the gradient was changed, and the chromatographic conditions were finally determined to achieve ideal separation efficiency and single peak width. Full Scan/dd-MS^2^ (full scan + automatic trigger secondary) was used as the scanning mode in the mass spectrometry. Through one injection, high-quality primary and secondary mass spectrometry data can be obtained simultaneously for qualitative results. According to the mass of the chemical constituents of the single herb reported in the literature, the scanning range m/z 70 ~ 1,050 was selected. After preliminary analysis by high performance liquid chromatography separation and mass spectrometry scanning in positive and negative ion modes, the response intensity of each component in positive and negative ion modes was significantly different. According to the physical and chemical properties of each component combined with its mass spectrometry response in different scanning modes, the ion mode with the best response was finally selected as the analysis condition, so that each analyte had a good response value and peak shape, which could meet the rapid and accurate qualitative requirements. This study shows that UHPLC-Q-Orbitrap / MS combined with Compound Discover 3.0 analysis software can achieve a simple and rapid structural identification of the chemical constituents of SFTOT. UHPLC-Q-Orbitrap/MS can realize the separation of unknown compounds in the preparation, complete the high-throughput workflow, and the determination of compounds is not limited by quantity. It can collect more comprehensive compound information, and can quickly and accurately screen and qualitatively analyze low-content potential compounds in complex systems.

The *in vitro* transdermal experiment is a method used to assess the skin penetration properties of drugs, employing isolated skin (animal, human, or artificial) in combination with specific analytical techniques. Several critical factors influence the experimental outcomes, including the choice of skin type, determination of the receiving medium, dosage settings, and control of the simulated temperature. In this experiment, we selected nude mouse skin based on its characteristics and suitability for the study. In choosing the receiving medium, we took into account the physical and chemical properties of the target drug, the biocompatibility of the medium with the skin, and its potential impact on skin barrier function. Previous studies have reported that a mixture of normal saline and varying concentrations of ethanol is commonly used as a transdermal receiving solution (27, 28). While ethanol can significantly enhance the transdermal rate and amount of non-water-soluble drugs, it may also over-accelerate drug permeation, leading to an inappropriate increase in the amount or rate of drug penetration. Moreover, the permeability of nude mouse skin is superior to that of human skin, further complicating the interpretation of results. Therefore, in this experiment, normal saline was selected as the transdermal receiving medium to avoid the potential over-enhancement caused by ethanol. To ensure appropriate drug delivery, we adhered to the principle of moderate dosing. If the dosage were too small, the drug could penetrate the skin completely before the end of the experiment. Conversely, if the dosage were excessive, uneven distribution and excessive residues could occur. Therefore, 1 g of SFTOT was chosen for use in this experiment. Additionally, since the skin surface temperature typically ranges around 32°C, we strictly controlled the water bath temperature to maintain a consistent range of (32 ± 1) °C to ensure the accuracy of experimental conditions.

In a transdermal drug delivery system, molecular weight and lipophilicity are critical factors that influence transdermal absorption efficiency. Drugs penetrate the skin via the intercellular space; however, when the molecular weight is too large, transmission resistance increases, and the drug is retained within the skin. It is generally accepted that drugs with a molecular weight of less than 500 Dalton (Da) have greater potential for efficient skin penetration (29). This study revealed that the molecular weight of the chemical components detected in the SFTOT transdermal receiving solution predominantly ranged from 80 to 470 Da, with over 95.3% of the components having a molecular weight below 400 Da. These characteristics suggest that the components exhibit excellent transdermal properties and can efficiently penetrate the skin barrier. Utilizing UHPLC-Q-Orbitrap/MS technology, we successfully identified 129 effective transdermal permeation components in SFTOT, providing crucial data for further investigation into the material basis and mechanism of SFTOT’s effects.

Among the 129 transdermal components, the network pharmacology and molecular docking analyses identified Luteolin, Kaempferol, Quercetin, 7-O-Methylluteolin, Apigenin, Ferulic acid, Palmitic acid, Sinapic acid, Shogaol, and Myristic acid as the core active compounds. These substances are the primary contributors to SFTOT’s therapeutic effects in treating COPD. They are mainly derived from CuYuanHua (CYH), XiXin (XX), JieZi (JZ), and GanJiang (GJ). Previous studies have demonstrated that these core active components possess various pharmacological effects relevant to COPD treatment. For example, Luteolin has been shown to inhibit macrophage phosphorylation and down-regulate pro-inflammatory factors, including interleukin-6 and tumor necrosis factor-*α* (30). Quercetin can significantly improve lung function in pulmonary fibrosis models by enhancing cell viability, reducing the production of inflammatory cytokines Tumor Necrosis Factor-alpha (TNF-α), Interleukin-6 (IL-6), Interleukin-8 (IL-8), and inhibiting apoptosis (31). Additionally, XiXin decoction can effectively reduce inflammatory ear swelling in mice (32), and Sinapic acid exerts anti-inflammatory effects by inhibiting the cyclooxygenase-2 and Nuclear Factor Kappa-light-chain-enhancer of Activated B cells (NF-κB) signaling pathways (33). Furthermore, Gingerol, derived from GJ, has been shown to reduce lung tissue injury by inhibiting the activation of the NF-κB pathway and to improve alveolar coagulation and fibrinolysis in rats with acute respiratory distress syndrome induced by lipopolysaccharide (34). The research presented above demonstrates that SFTOT contains various effective components that alleviate cough and asthma-related diseases by reducing the inflammatory response. These findings offer significant insight for further investigation into SFTOT’s therapeutic potential.

Protein–protein interaction (PPI) network analysis further suggests that SFTOT may exert its therapeutic effects in COPD through key targets, including Tumor necrosis factor (TNF), Albumin.

(ALB), AKT Serine/Threonine Kinase 1(AKT1), Epidermal Growth Factor Receptor (EGFR), and Caspase-3 (CASP3). TNF-*α*, a small molecule protein secreted by macrophages, plays a critical role in both the local and systemic inflammatory responses associated with COPD. Elevated levels of TNF-α in COPD patients exacerbate inflammation and damage alveolar epithelial cells (35), making it a crucial mediator in the regulation of COPD inflammation. ALB, the most abundant protein in human plasma, is often found at decreased levels in COPD patients, reflecting a disruption in its normal function during the disease (36). AKT1, a member of the AGC protein kinase family, is integral in regulating cell growth, division, and apoptosis inhibition. Its multifaceted role may influence the cellular mechanisms central to the pathological processes of COPD (37). EGFR, a key regulator of cellular activities, is associated with increased airway mucus secretion. SFTOT may help reduce airway mucus hypersecretion in COPD by modulating EGFR expression (38). CASP3, involved in the regulation of airway smooth muscle cell apoptosis through the Polo-like Kinase 1 - Caspase-3 (PLK1-CASP3) pathway, is another vital target for COPD treatment (39).

To further elucidate the underlying mechanisms of SFTOT, Kyoto Encyclopedia of Genes and Genomes (KEGG) pathway enrichment analysis was conducted on the component-disease intersection targets. The results of the analysis were primarily enriched in key signaling pathways, including Advanced Glycation End-products - Receptor for Advanced Glycation End-products (AGE-RAGE), TNF, PI3K/protein kinase B (Akt) signal transduction pathway (PI3K-Akt), and mitogen-activated protein kinase (MAPK). Among these, RAGE, TNF, and the MAPK signaling pathways are closely associated with the inflammatory response and oxidative stress (40–42). The PI3K-Akt pathway, a crucial intracellular signaling cascade, regulates various cellular biological processes and the cell cycle. By influencing downstream effector molecules involved in apoptosis, transcription, translation, metabolism, and angiogenesis, the PI3K-Akt pathway plays a pivotal role in COPD airway remodeling (43, 44). These findings suggest that SFTOT may exert its therapeutic effects through the modulation of multiple signaling pathways, offering a novel perspective and potential strategy for COPD treatment.

## 5 Conclusion

Research on the material basis of Traditional Chinese Medicine (TCM) compounds is essential for ensuring quality control and safety, playing a key role in the modernization of TCM. In this study, Shufeitie ointment (SFTOT) was selected as the research object. Using the high sensitivity and precision of liquid chromatography-mass spectrometry (LC–MS), we conducted the first qualitative analysis of the transdermal permeation components, identifying the chemical constituents. This laid a solid scientific foundation for subsequent formulation optimization and comprehensive quality control research. Additionally, by employing network pharmacology and molecular docking techniques, we made the first molecular-level predictions of the pharmacodynamic mechanisms of SFTOT. We also identified key quality markers associated with its efficacy, providing valuable insights for improving the quality standards of SFTOT.

However, due to the complexity of traditional Chinese medicine’s chemical composition, several limitations exist in this study. These include the inability to identify isomers and potentially new components generated in the prescription. While we employed high-resolution LC–MS, the analysis did not cover volatile components. Although we performed preliminary molecular docking validation, experimental verification is still lacking. As a result, some discrepancies between the predicted results and the actual outcomes may exist. Addressing these limitations, we plan to conduct further research in the future to fill these gaps and refine the findings.

## Data Availability

The original contributions presented in the study are included in the article/Supplementary material, further inquiries can be directed to the corresponding authors.

## Footnotes

^1^ www.mzcloud.org

^2^ www.chemspider.com

^3^ http://swisstargetprediction.ch/

^4^ https://lilab-ecust.cn/pharmmapper/index.html

^5^ https://sea.bkslab.org/

^6^ https://www.omim.org/

^7^ https://go.drugbank.com/

^8^ https://www.genecards.org/

^9^ https://www.uniprot.org/

^10^ https://string-db.org/

^11^ https://david.ncifcrf.gov/

^12^ https://cytoscape.org/

^13^ https://www.rcsb.org/

^14^ https://autodock.scripps.edu/
