# Supplementary material for: Potential active ingredients and mechanisms of Shufeitie ointment in the treatment of chronic obstructive pulmonary disease by integrating transdermal chemistry and network pharmacology
Source: Front Med (Lausanne). 2025 Jul 8;12:1605372. doi: 10.3389/fmed.2025.1605372 (PMC12279505; doi:10.3389/fmed.2025.1605372)
Supplement: Supplementary file 1 [file Table_1.docx]

***Supplementary materials***

Table S1 Abbreviations

ALB Albumin

BC Betweenness centrality

BP Biological process

CASP3 Caspase-3

CC Closeness centrality/Cell composition/cellular component

COPD Chronic Obstructive Pulmonary Disease

CYH CuYuanHua

CYHS CuYanHuSuo

EGFR Epidermal growth factor receptor

ESI Electrospray ionization

GJ GanJiang

GO Gene Ontology

IVPT In Vitro Permeation Test

JM JiaoMu

JZ JieZi

KEGG Kyoto Encyclopedia of Genes and Genomes

MF Molecular function

PPI Protein-protein interaction

RG RouGui

SFTOT Shufeitie ointment

TCM Traditional Chinese Medicine

TNF Tumor necrosis factor

UHPLC-Q-Orbitrap/MS Ultra-high-performance liquid chromatography-quadrupole/electrostatic field orbitrap high-resolution mass spectrometry

XX XiXin

Table S2 GO enrichment analysis of the top 10 pathways

| **Term** | **GeneRatio** | **P-value** | **Count** | **Class** |
| --- | --- | --- | --- | --- |
| response to hypoxia | 14.41441441 | 4.20E-28 | 32 | BP |
| response to xenobiotic stimulus | 16.21621622 | 1.58E-26 | 36 | BP |
| positive regulation of MAPK cascade | 10.36036036 | 4.01E-17 | 23 | BP |
| negative regulation of apoptotic process | 16.21621622 | 5.11E-17 | 36 | BP |
| inflammatory response | 14.41441441 | 2.86E-16 | 32 | BP |
| positive regulation of MAP kinase activity | 7.657657658 | 3.18E-16 | 17 | BP |
| response to lipopolysaccharide | 9.459459459 | 4.38E-16 | 21 | BP |
| proteolysis | 14.41441441 | 1.33E-15 | 32 | BP |
| protein phosphorylation | 13.96396396 | 4.04E-15 | 31 | BP |
| extracellular matrix disassembly | 5.855855856 | 9.49E-14 | 13 | BP |
| extracellular region | 31.98198198 | 1.19E-17 | 71 | CC |
| plasma membrane | 53.15315315 | 2.61E-17 | 118 | CC |
| membrane raft | 10.36036036 | 3.31E-14 | 23 | CC |
| receptor complex | 9.90990991 | 4.38E-14 | 22 | CC |
| extracellular space | 27.47747748 | 6.55E-14 | 61 | CC |
| cell surface | 15.31531532 | 1.39E-13 | 34 | CC |
| integral component of plasma membrane | 21.62162162 | 5.96E-12 | 48 | CC |
| macromolecular complex | 14.41441441 | 1.86E-11 | 32 | CC |
| mitochondrion | 20.72072072 | 1.04E-10 | 46 | CC |
| extracellular exosome | 26.12612613 | 3.01E-10 | 58 | CC |
| enzyme binding | 19.36936937 | 1.12E-28 | 43 | MF |
| identical protein binding | 32.43243243 | 4.78E-22 | 72 | MF |
| endopeptidase activity | 7.657657658 | 7.23E-16 | 17 | MF |
| serine-type endopeptidase activity | 9.90990991 | 5.44E-15 | 22 | MF |
| RNA polymerase II transcription factor activity, ligand-activated sequence-specific DNA binding | 5.855855856 | 1.04E-12 | 13 | MF |
| heme binding | 8.108108108 | 3.41E-12 | 18 | MF |
| protein serine/threonine/tyrosine kinase activity | 11.71171171 | 8.25E-11 | 26 | MF |
| peptidase activity | 6.306306306 | 2.78E-10 | 14 | MF |
| protein binding | 85.13513514 | 4.29E-10 | 189 | MF |
| metalloendopeptidase activity | 6.306306306 | 6.30E-10 | 14 | MF |

Table S3 KEGG enrichment analysis of the top 20 pathways

| **Term** | **GeneRatio** | **P-value** | **Count** |
| --- | --- | --- | --- |
| Proteoglycans in cancer | 12.16216216 | 1.11E-12 | 27 |
| Human T-cell leukemia virus 1 infection | 12.61261261 | 1.09E-12 | 28 |
| Ras signaling pathway | 13.06306306 | 7.45E-13 | 29 |
| MAPK signaling pathway | 14.86486486 | 3.16E-13 | 33 |
| PI3K-Akt signaling pathway | 16.21621622 | 2.85E-13 | 36 |
| Hepatitis B | 11.26126126 | 2.79E-13 | 25 |
| Chagas disease | 9.459459459 | 1.30E-13 | 21 |
| TNF signaling pathway | 9.90990991 | 1.14E-13 | 22 |
| Pancreatic cancer | 8.558558559 | 7.07E-14 | 19 |
| Osteoclast differentiation | 10.81081081 | 4.16E-14 | 24 |
| Prostate cancer | 9.90990991 | 3.66E-15 | 22 |
| Fluid shear stress and atherosclerosis | 11.71171171 | 8.35E-16 | 26 |
| Chemical carcinogenesis - receptor activation | 13.96396396 | 8.28E-16 | 31 |
| Chemical carcinogenesis - reactive oxygen species | 14.41441441 | 3.77E-16 | 32 |
| Diabetic cardiomyopathy | 13.96396396 | 2.12E-16 | 31 |
| Relaxin signaling pathway | 11.71171171 | 1.14E-16 | 26 |
| Kaposi sarcoma-associated herpesvirus infection | 13.96396396 | 5.86E-17 | 31 |
| AGE-RAGE signaling pathway in diabetic complications | 10.81081081 | 3.70E-17 | 24 |
| Lipid and atherosclerosis | 15.76576577 | 1.92E-19 | 35 |
| Pathways in cancer | 27.47747748 | 2.53E-26 | 61 |
